# Integrated Decentering: Validating an Automated Measure of Decentering Expression Across Seven Independent Samples

**DOI:** 10.21203/rs.3.rs-10410851/v1

**Published:** 2026-08-14

**Authors:** Tamas Madl, Sara W. Lazar

**Affiliations:** 1 Austrian Research Institute for Artificial Intelligence (OFAI); University of Vienna; 2 Massachusetts General Hospital

**Keywords:** Decentering, Cognitive defusion, Mindfulness, Construct validity, Natural language processing, Psychological measurement

## Abstract

**Objectives.:**

Decentering—relating to one’s thoughts and emotions as passing mental events rather than being fused with them—is difficult to measure at scale. We test whether Integrated Decentering (ID), an automated measure of the decentering expressed in short reflective text, shows construct validity as one fixed scoring model.

**Methods.:**

A purpose-trained language-model ensemble scored texts from seven independent samples (self-report, community, essay, diary, and online-argument samples, and two contemplative-practice cohorts). One cohort was a post-hoc secondary analysis of a registered randomized trial; ID was not a prespecified trial outcome. Aggregate-level analyses tested convergent, criterion, discriminant, incremental, reliability, and practice-linked validity, controlling for text length.

**Results.:**

ID converged with established decentering scales (EQ-D *r* = .36, 95% CI [.20, .50], *n* = 124; MPoDT *r* = .41 [.09, .65], *n* = 35); no prompted general-purpose model, frontier included, reproduced the EQ-D convergence. ID predicted empathic concern (*r* = .40 [.36, .44]), wise-reasoning search-for-resolution (*β* = .18 [.05, .30]), and value–behavior coherence (*r* = .33 [.24, .41]; Δ*R*^2^ = .027 beyond the Big Five). Predicted nulls held (adjacent wise-reasoning subscales, rumination, negative affect), and length-matched impersonal argument showed no positive discrimination (AUC = .45 [.40, .49]). Aggregates were stable over 12 months (*r* = .69 [.52, .81]). ID distinguished practitioners from matched non-practitioner controls (meditation *g* = 1.83 [1.08, 2.89]; yoga *g* = 1.38 [0.68, 2.29]); in a post-hoc non-randomized exposure analysis, reported sitting practice tracked 24-month gains (*g* = .80 [0.13, 1.56]). ID overlapped with an expert developmental score, as theory predicts, yet predicted decentering-relevant self-report beyond it.

**Conclusions.:**

One fixed scoring model transferred unchanged across all seven samples: ID offers a scalable measure of decentering expression in reflective text for contemplative science and other fields concerned with metacognitive distance.

**Trial registration.:**

ClinicalTrials.gov NCT02628548; registered 11 December 2015.

## 1 Introduction

### 1.1 Decentering and the problem of measuring it

A long contemplative and clinical lineage holds that suffering often stems less from thoughts and feelings themselves than from how one relates to them. Decentering is the metacognitive shift from being fused with mental contents—treating thoughts, emotions, and self-concepts as the self—to holding them as transient, constructed mental events. Bernstein, Hadash, and Fresco [Bernstein et al., 2015] frame this fusion-to-defusion process as a unitary metacognitive construct that spans contemplative and clinical traditions: decentered awareness in mindfulness-based cognitive therapy, cognitive defusion in acceptance and commitment therapy, and the broader mindfulness literature. The same shift is central to Shapiro and colleagues’ “reperceiving” account of the mechanisms of mindfulness [Shapiro et al., 2006]—one of several labels (decentering, cognitive defusion, reperceiving, self-distancing) under which the contemplative and clinical literatures have converged on a common metacognitive shift.

Self-report operationalizations of decentering mediate the effects of mindfulness-based interventions on clinical outcomes [Gu et al., 2015, van der Velden et al., 2015], and the same capacity is implicated well beyond contemplative practice—in emotion regulation, empathy, wise reasoning about conflict, and the perspective-taking that supports prosocial action. Experimentally, a decentered stance toward imagined stressful events reduces their affective impact [Lebois et al., 2015]. Decentering is thus a candidate mechanism of unusually broad reach, which makes its measurement consequential.

Two measurement channels dominate. The first is self-report: trait scales such as the Experiences Questionnaire (EQ) [Fresco et al., 2007] and the Metacognitive Processes of Decentering–Trait scale (MPoDT) [Hanley et al., 2020] (and, for the closely related ACT construct of cognitive fusion, the Cognitive Fusion Questionnaire [Gillanders et al., 2014]) are well validated and clinically useful. But they inherit the familiar limits of introspective report: sensitivity to demand characteristics, dependence on the respondent’s metacognitive accuracy, and a state-versus-trait distinction that varies by instrument and is easily overlooked (the Toronto Mindfulness Scale [Lau et al., 2006] targets state, the EQ and MPoDT trait). More acutely in contemplative populations, self-report also presupposes the very capacity it assesses: rating one’s own decentering requires meta-awareness of one’s fusion. Practitioners with heightened meta-awareness may under-rate their decentering precisely because they see their fusion more clearly, while novices over-rate theirs—a reference-group bias documented in mindfulness self-report [Grossman, 2008, Van Dam et al., 2018] that language-based measurement does not share in the same form, though (as we note below) it carries biases of its own.

The second channel, specific to contemplative research, is practice exposure: lifetime or in-trial practice hours used as a proxy for how much decentering an intervention has instilled. Yet exposure indexes delivery, not internalization. Home-practice–benefit correlations in mindfulness trials average only *r* ≈ .26 [Parsons et al., 2017], dose–response meta-regressions find no robust practice effect on distress outcomes [Strohmaier, 2020], and in long-term meditators lifetime practice accounts for a modest, non-linear share of wellbeing variance [Bowles et al., 2022]. At matched exposure, some practitioners show durable change and others do not. Both capture internalized decentering only partially: one asks people to report a capacity they may not observe accurately, the other counts hours rather than their effect.

### 1.2 Language as a complementary measurement channel

Reflective language offers a third channel. How a person construes a charged situation in their own words—fused with a thought, emotion, or identity, or holding it as partial, transient, and constructed—carries information about decentering that neither a Likert rating nor an hour count contains [Lanning et al., 2018]. Naturalistic text reduces dependence on the respondent’s metacognitive accuracy and is less transparent to demand than a questionnaire item. Until recently the obstacle was scale: extracting this signal required expert human coding, as in the interview-based Measure of Awareness and Coping in Autobiographical Memory [Teasdale et al., 2002]—slow, costly, and hard to standardize. Language models now make it feasible to score reflective constructs from short free-text passages automatically and reproducibly, opening this channel to large, multi-sample validation.

Automated text methods have so far indexed the neighbouring construct of psychological self-distancing through surface lexical markers such as first-person-pronoun and present-tense rates [Nook et al., 2017, Kross and Ayduk, 2017]; ID instead scores decentering directly with a construct-trained model rather than relying on such markers, and is not reducible to them: a validated LIWC-22 distancing index is near zero with the criteria ID predicts in the two large samples (WASSA, Grossmann; Supplementary Materials S15). Prior language-model work in psychology has largely fit a supervised model to a single labeled outcome in a single dataset; we instead validate one fixed, construct-targeted scorer as a measurement instrument: across samples, against discriminant boundaries, and incrementally over alternative accounts.

### 1.3 Integrated Decentering

We introduce Integrated Decentering (ID), a measure that scores the decentering expressed in short reflective English text on a continuous scale. ID is a measurement method for the existing decentering construct [Bernstein et al., 2015], not a proposal for a new construct: the qualifier “integrated,” explained below, describes the level at which the measure is validated, not a new theoretical entity.

To make the construct concrete, consider two first-person responses to the sentence stem “When I am criticized…” (public-domain WUSCT-tradition corpus [Lanning et al., 2018], quoted verbatim):

*More decentered:* “I may feel hurt initially, but later try to honestly judge the validity of the criticism (diffidult! [sic]).”

*More fused:* “I often agree with what has been said about me and become sad (once in a while mad).”

Both are comparable in length and name a negative reaction to the same stem; what differs is the stance toward that reaction—whether it is held as a passing mental event and examined, or taken as simply true of the self. These illustrate the construct, not ID’s scores: ID is validated on per-person aggregates, not single completions.

ID is produced by a purpose-built model ensemble (Section 2); here we note only the properties that bear on interpretation. ID is not a general-purpose chatbot prompted to rate passages; it is a fixed, purpose-trained scorer, fine-tuned with a contrastive objective to judge which of two reflective texts expresses more decentering. ID scores *texts, not people*: a single passage’s score is an estimate of the decentering expressed in that passage, and the validated unit is the aggregate (per person or condition for sentence completions; per text or event for essays and diaries), not the isolated single completion.

We call the measure *Integrated* Decentering because it indexes decentering not as a single act but as it has become woven into a person’s habitual meaning-making—metacognitive distance integrated into how experience is spontaneously construed and narrated. The training signal was built from reflective sentence-completion material in the Loevinger/WUSCT tradition and its adult-developmental extensions [Lanning et al., 2018, Madl and Cook-Greuter, 2026]; that material is a source of reflective text and contrastive structure, not the definition of the target. Accordingly, we do not treat ID as an automated developmental-stage rating, and we validate it directly against decentering criteria, not the developmental scoring from which its training pairs were drawn.

ID measures decentering as *expressed in language*, not decentering as lived. A contemplatively mature person may describe experience in plain, unmarked speech; a psychologically literate writer may produce decentered phrasing without the corresponding shift in perspective. The anti-shortcut training, text-length controls, and the null result on length-matched impersonal argument (Section 4.5) mitigate but cannot eliminate this gap. ID is best understood as one imperfect behavioral trace—a third channel alongside, and convergent with, the first-person perspective that self-report uniquely provides—not a replacement for it, nor a measure of contemplative attainment.

### 1.4 Validation logic

A new measure earns its interpretation not from any single correlation but from a coherent convergent-and-discriminant pattern across methods [Cronbach and Meehl, 1955, Campbell and Fiske, 1959]. We therefore draw on multiple sources of validity evidence for ID across seven heterogeneous samples, each assigned a prediction the construct should generate (Figure 1):

- **Convergent.** ID should correlate with established self-report decentering scales (EQ-D, MPoDT).
- **Nomological / criterion.** ID should predict theoretically adjacent outcomes—empathic concern, wise reasoning about conflict, and coherence between stated values and reported behavior—while remaining null on adjacent constructs the theory does not implicate.
- **Discriminant / boundary.** ID should show no positive discrimination in an impersonal, argumentative register where the construct does not apply, once text length is matched.
- **Practice-linked.** ID should be elevated in contemplative practitioners relative to controls, more so for the practice that directly trains decentering, and should track meditation exposure over time (the exposure test is post hoc; Section 3.2).
- **Incremental.** ID should carry information beyond text length and verbal ability, beyond broad personality, and beyond a human expert’s developmental score for the same protocols. This is the closest developmental benchmark, since it taps a maturity-relevant axis shared with ID yet is scored independently.

No single sample resolves the construct claim; the samples’ principal weaknesses differ across the design; and no single confound we could test can jointly produce the pattern—the argument is cumulative. The claim is correspondingly falsifiable: convergence indistinguishable from a generic verbal or maturity factor, criterion associations lacking the predicted specificity, or above-chance discrimination in the length-matched argumentative register would each count against it. Each alternative account or near-neighbor benchmark is addressed by a different cell of the design (Table 3): text length and verbal ability, broad personality, generic positivity and self-reflection, surface linguistic distancing, a human expert’s developmental score, and zero-shot LLM scoring (prompting a general-purpose model, without task-specific training).

The evidence is tiered: the convergent, nomological, behavioral/incremental, and discriminant tests form the core of the validation; the practice-linked results test sensitivity to contemplative exposure; and the neuroimaging analysis is reported as an exploratory extension. That extension shows ID recovers, automatically from text, a developmentally gated pattern of posterior-cingulate connectivity previously reported for this cohort. Throughout, we delimit ID’s scope: a scalable research instrument for decentering expression, not a diagnostic, neural marker, or developmental-stage measure.

Practically, because ID scores text that studies already collect, any trial or cohort with open-ended reflections, practice diaries, or exit interviews can rescore that existing text for a decentering measure at no additional participant burden—an applied payoff we return to in the Discussion.

## 2 The Integrated Decentering scorer

### 2.1 What the scorer estimates

ID is a continuous scorer of the decentering expressed in short reflective English text. Given a passage, it returns a value on a 0–9 scale, where higher scores indicate stronger textual evidence of stepping back from one’s thoughts, affects, identities, or perspectives and holding them as transient, constructed events. The scorer estimates a property of the *text*, not a stable attribute of the writer; aggregates—per-person or per-condition means for sentence completions, and per-text or per-event scores for essays and diaries—are the unit at which validity is established and at which the analyses below are reported. Full architectural and training detail is given in Supplementary Materials S1; we summarize here only what is needed to interpret the results.

### 2.2 Architecture and training

Conceptually, the scoring model was trained much as one might train a human rater. It was shown many thousands of pairs of reflective texts—in each pair, one expressing more decentering than the other—and learned to judge which of the two expresses more. Many of these pairs were matched on developmental stage and on text length, so the model could not tell the two apart by overall maturity or by verbosity and had to rely on the decentering contrast itself (we call these “anti-shortcut” pairs). ID still shares a maturity-relevant axis with expert developmental scoring but does not reduce to it, as we quantify later. The final measure averages the judgements of two independently trained models, analogous to averaging two trained raters. Its parameters were fixed and versioned before any validation text was scored; no dataset-specific tuning, prompting, or calibration occurred at any point.

The technical implementation—an equal-weight ensemble of two independently trained low-rank (LoRA) adapters over open-weight Qwen base models—is detailed in Supplementary Materials S1.

Rather than regressing text onto a developmental-stage label, each member was trained with a **contrastive Bradley–Terry objective** [Bradley and Terry, 1952] on pairs of passages, predicting which of two texts expresses more decentering. The training signal was derived from reflective sentence-completion material in the Loevinger/WUSCT tradition and its adult-developmental extensions [Lanning et al., 2018, Madl and Cook-Greuter, 2026], using a decentering-aligned indicator from that upstream work as a deliberately imperfect, theory-motivated proxy. The length-matched pairs were of three kinds; the up-weighted **within-stage pairs** (same developmental stage, different decentering proxy) suppress “stage shortcut” learning, so the scorer tracks reflective distance rather than a coarse developmental bucket (Supplementary Materials S1). ID and expert developmental scoring nonetheless share a maturity-relevant axis—the subject–object differentiation that decentering and ego development are theorized to hold in common—so the two agree substantially; this expected overlap reflects that shared axis and same-protocol scoring rather than stage recovery, and is not by itself evidence of validity (Section 3.2). The construct claim therefore rests not on the size of that agreement but on differential prediction—the non-developmental criteria below (empathy, wise reasoning, value–behavior coherence), and the fact that ID still predicts decentering-relevant self-report after the expert score is accounted for (Section 4.5).

### 2.3 Optional eligibility filtering

Decentering is meaningfully scoreable only when a passage contains first-person or self-relevant material the writer could identify with—a belief, value, feeling, identity, or perspective. Purely factual text carries no such signal; any score on it is dominated by length. For heterogeneous inputs we therefore use two pre-specifiable eligibility filters that operationalize this precondition: a deterministic *mechanical* filter (first-person, belief, or affect markers) and a more permissive *LLM* filter (a separate model used only to judge eligibility, not the ID scorer; Supplementary Materials S2). Filtering also removes unscoreable, length-only completions from short sentence-completion data; this matters most in small samples. We report the unfiltered view alongside as a sensitivity check and treat filtered estimates as primary only for the mixed-register essay corpus. Filter views are subset operations on the same per-text scores (Supplementary Materials S2). Because the sentence-completion stems are shared across participants, we also verified that scoring the completion alone rather than the full stem-plus-completion leaves convergent estimates materially unchanged, ruling out a shared-stem artifact (Supplementary Materials S4).

### 2.4 Scope and boundary conditions

ID is validated for written, reflective English text of roughly sentence-to-paragraph length; it is strongest for self-relevant reflective material and most defensible when aggregated. It has not been validated for other languages or non-reflective registers, and should not be used for decisions about identifiable individuals or to infer a person’s mindfulness, wisdom, mental health, or developmental stage. One boundary is itself a validation target: on impersonal text that is neither self-relevant nor emotionally engaged (e.g. persuasive argument about an external topic) the scorer can behave as a length proxy (Section 4.5), so it should not be applied to such content as if it measured decentering.

The training material is disjoint from every validation sample at the participant and response level: none of the validation data evaluated here entered training (the sentence-completion prompts are the standard shared format, but no training individual or response appears in the validation samples). A distinct concern is *pretraining* exposure: some validation samples (WASSA, CMV, MTurk) are public and could occur in the base models’ pretraining data. Three observations bound this concern: the task is judging a passage, not recalling it, and no criterion labels are paired with text in any public release in scoreable form; the untuned base ensemble—with identical pretraining—carries no convergent signal (Supplementary Materials S15); and the convergent, reliability, and practice-linked evidence comes from private clinical cohorts that are not part of any public corpus.

## 3 Validation datasets and analytic strategy

### 3.1 Participants and measures, organized by validation role

We validate ID across seven independent samples (Table 1), each carrying one or more validation roles. The samples are deliberately heterogeneous in population, text genre, and criterion type, and were not collected for ID; several predate the scorer entirely. Brief descriptions follow; full per-sample procedures, measures, and sample characteristics are in the Supplementary Materials.

#### Convergent.

The **MGH aging** cohort (below) provides the larger, better-powered convergent anchor: the 11-item Experiences Questionnaire decentering subscale (EQ-D) [Fresco et al., 2007], with *n* = 124 holding both ID and EQ-D. The **MPoDT** sample, collected by the authors via Prolific, comprises 35 adults who completed sentence-completion stems and the Metacognitive Processes of Decentering–Trait scale (MPoDT) [Hanley et al., 2020], an established text-relevant decentering self-report; it serves as a smaller corroborating anchor whose subscale structure additionally probes construct coverage (Section 4.2).

#### Nomological / criterion.

**WASSA** [Buechel et al., 2018] is a corpus of 1,860 first-person essays written in response to news articles, each rated for empathic concern and personal distress; decentering should track the compassionate (non-fused) component of empathy. The **Grossmann** conflict-diary sample [Grossmann et al., 2021] contains 424 first-person accounts of interpersonal conflict (298 participants), each coded by trained raters on five wise-reasoning subscales [Brienza et al., 2018]; decentering should predict search-for-resolution and perspective-taking but not the three adjacent subscales (compromise, recognition of limits, expectation of change). The **MTurk** community sample [Fournier et al., 2022] comprises 446 middle-aged adults with sentence completions, the Big Five Inventory–2 (BFI-2), and a value–behavior coherence index computed across three close relationships.

#### Discriminant / boundary.

The **CMV** corpus [Tan et al., 2016] pairs, for each Reddit r/ChangeMyView post, one argument that changed the poster’s view with one that did not (4, 311 pairs). Persuasive success in this impersonal argumentative register should not reflect decentering once text length is matched.

#### Practice-linked and incremental.

The **MGH neuroimaging** cohort [Gard et al., 2014, Gard et al., 2015, Singleton et al., 2021] is a secondary analysis of 45 adults—meditation practitioners (*n* = 14), yoga practitioners (*n* = 16), and matched controls (*n* = 15)—with sentence completions, a behavioral battery (incl. Perceived Stress Scale (PSS), Quality of Life Inventory (QOLI), Self-Compassion Scale (SCS), American National Adult Reading Test (AMNART) verbal IQ), resting-state fMRI (posterior cingulate cortex [PCC] seed connectivity), and an expert human developmental rating of the same protocols. The **MGH aging** cohort is a post-hoc secondary analysis of a randomized trial of meditation-naive older adults [Sevinc et al., 2021], prospectively registered on 11 December 2015 (ClinicalTrials.gov NCT02628548). The registry records 175 enrolled in the overall study, the primary report records 146 randomized (72 mindfulness, 74 cognitive-fitness control), and the present secondary dataset contains sentence-completion text for 143 participants at one or more analyzed waves; these are different recruitment, randomization, and data-availability denominators (full accounting in Supplementary Materials S10). Sentence completions were available at baseline, 12, and 24 months, with practice follow-up available for a subset. This single cohort provides three of the results reported below—the EQ-D convergent correlation, the 12-month retest stability, and the 24-month dose–response—which are therefore not independent: they are separate analyses (different waves and subsamples) of one study. Neither dataset was collected with ID in mind, and neither set of ID analyses was pre-registered; ID was not a prespecified trial outcome.

### 3.2 Analytic strategy

All analyses use per-person (or, for the essay/argument corpora, per-text) ID aggregates. Because ID is text-derived, every model controls for verbosity (log word count) and, where relevant, language covariates; convergent and criterion associations are Pearson or partial correlations net of these controls. Group differences use Welch’s *t* with Hedges’ *g* (a small-sample-corrected analogue of Cohen’s *d*), reported with exact noncentral-*t* 95% confidence intervals. Criterion and connectivity models use ordinary least squares with HC3 heteroskedasticity-robust standard errors; the Grossmann diary models, which contain multiple events per participant, use subject-clustered standard errors. Incremental validity is assessed by hierarchical Δ*R*^2^ (e.g., ID added to a model already containing the Big Five). The discriminant boundary uses paired and length-matched area-under-the-curve (AUC). Headline effects are reported with 95% confidence intervals; secondary, filtered, and covariate-adjusted estimates are reported with exact *p*-values (reproducible script 25_headline_cis.py).

ID is also compared against a human expert’s Total Weighted Score (TWS)—a cumulative expert score of ego development—for the same sentence-completion protocols, scored independently of the language model and previously used on the neuroimaging cohort by Singleton et al. [Singleton et al., 2021]. A substantial ID–TWS correlation is expected for two compounding reasons: the two tap a shared, maturity-relevant axis (the subject–object differentiation at the core of ego development [Kegan, 1982, Cook-Greuter, 1999]), and both are scored from the same protocols, so shared text properties (e.g., articulateness) inflate their agreement; the scorer’s contrastive training nonetheless discourages stage-bucket shortcuts (Section 2). TWS is a *cumulative* score, not a developmental stage: stage is assigned to a protocol by threshold (cut-off) rules. Because the same total can arise from different response profiles, the score-to-stage map is many-to-one, so agreement with TWS is uninformative about whether ID recovers discrete stage. Consistent with this, ID correlates with discrete stage well below TWS (*r* = .69 versus *r* = .95); squaring these, stage accounts for about 48% of ID’s variance against roughly 90% of TWS’s, so the within-stage training attenuated, rather than eliminated, the stage association (Supplementary Materials S9). We test whether ID carries information beyond TWS using Williams’ *t* for the difference between two dependent correlations sharing a variable (*df* = *n*−3; eq. 7 in [Steiger, 1980]) and joint models partitioning their unique contributions (Δ*R*^2^); we report the human-coded TWS throughout the main text and a model-derived maturity score only as a robustness check (Supplementary Materials S14).

For theory-specified tests with a priori directional hypotheses (convergent correlations, known-groups separation, the criterion associations, the discriminant boundary), we use *α* = .05 uncorrected; for exploratory sets of correlations within a sample we apply Benjamini–Hochberg false-discovery-rate (FDR) control. For completeness, the primary directional family (convergent, criterion, and known-groups tests, *m* = 7) also survives Benjamini–Hochberg FDR control at *q* = .05, with a largest surviving *p* of .007 (Grossmann Search-for-Resolution; script 28_robustness_followups.py). The exploratory neural moderation is the one exception: its regions and developmental split are hypothesis-constrained from prior work on this cohort, and it is reported uncorrected and explicitly flagged as not surviving correction. Every regression effect was checked for robustness with leave-one-out and Cook’s-distance diagnostics, and every filtered analysis against the unfiltered view; both are reported in the supplement. Effect sizes are interpreted against individual-differences norms in which *r* ≈ .10, .20, and .30 are small, typical, and relatively large, with *r* ≥ .50 rare [Gignac and Szodorai, 2016]. Verified, reproducible code for every number in this paper is provided (see Data and Code Availability).

#### Analysis provenance.

Because neither contemplative cohort was collected for ID and none of these analyses was preregistered, we distinguish analyses by evidential status. *Theory-guided primary tests*—the convergent correlations, the criterion associations (empathy, wise reasoning, value–behavior coherence), the known-groups separation, and the length-matched discriminant boundary—each test an a priori directional hypothesis. *Pre-specifiable filter logic*: the two eligibility filters operationalize a stated scoreability precondition and are fixed by register rather than tuned to outcomes (Section 2), with the unfiltered view always reported alongside. *Robustness and sensitivity checks*—filter-view comparisons, verbosity and demographic adjustments, cross-validated increments, and leave-one-out diagnostics—probe whether a primary result depends on an analytic choice. *Post-hoc exposure analyses*: the 24-month dose–response is non-randomized and conditioned on who was reached and chose to practice, and is labelled as such throughout. Within it we treat the categorical any-sitting-practice contrast (*g* = 0.80) as the primary exposure estimate, with the continuous frequency association (*ρ* = .36) and the joint practice-vs-arm model as robustness checks on the same exposure rather than independent tests. *Exploratory neural analyses*: the connectivity moderation is hypothesis-constrained from prior work on this cohort but reported uncorrected and flagged as not surviving correction. We report all of these, not only the confirmatory subset.

## 4 Results

Results are organized by validity type rather than by sample, each subsection assembling evidence across whatever samples bear on it. Table 2 and Figure 2 summarize every result on one effect-size axis. The argument is cumulative; no single effect carries the construct claim.

### 4.1 Measurement behavior and reliability

ID scores were approximately normally distributed in every sample and correlated modestly with text length (Spearman *ρ* ≈ .2–.3 across samples). Part of this is expected on substantive, not only psychometric, grounds: holding a thought or feeling as a transient, examinable mental event tends to take more words than asserting it as simple fact, so decentering plausibly co-occurs with somewhat fuller elaboration of inner experience rather than length being purely a nuisance. We nonetheless control for verbosity (log word count) throughout; this is conservative—it removes construct-relevant along with nuisance variance—so the criterion associations that survive it are, if anything, understated.

A single completion is a weak index of the person (single-stem intraclass correlation ICC(1, 1) ≈ .05–.09), as expected for one short stem, but averaging over a participant’s stems (mean 32, 18, and 36 per person) yielded reliable aggregate scores (ICC(1, *k*) = .75 in the neuroimaging cohort, .65 in the MPoDT sample, and .66 per person-wave in the aging cohort; split-half Spearman–Brown *r* = .73; single-stem reliabilities in Supplementary Materials S1). ID is therefore analyzed and reported at the per-person or per-condition level in these sentence-completion cohorts. The two ensemble members agreed on training-format text (*r* ≈ .93) but diverged enough across the validation battery to justify averaging (Supplementary Materials S1).

In the randomized aging cohort, the active-control arm (assigned games and puzzles, not meditation) gives a 12-month stability estimate: Pearson *r* = .69 (95% CI [.52, .81], *n* = 56), comparable at 24 months and in the range of established trait measures. Because some control participants practiced on their own (Section 4.6), this reflects the stability of a partly practice-exposed sample rather than a pure intervention-free estimate. This stability is the first evidence that ID indexes a *consolidated* disposition rather than a momentary state: the same individuals received similar scores a year apart, even as the measure remained responsive to sustained practice (Section 4.6). Aggregate ID is also robust to the choice of eligibility filter; filter views change convergent correlations by ≤ .06 and never reverse a conclusion (Supplementary Materials S2, S14).

### 4.2 Convergent validity

A valid decentering measure should converge with established self-report decentering scales; ID converged with both. The larger, better-powered anchor is the EQ-D: in the aging cohort, ID correlated with the EQ-D subscale at *r* = .36 (95% CI [.20, .50], *n* = 124, *p* < .001), holding beyond verbosity on the scoreable view (partial *r* = .33) and stable across filter views (*r* = .30–.33). The smaller MPoDT sample corroborates: per-person ID correlated with the MPoDT trait-decentering composite at *r* = .41 (95% CI [.09, .65]) across all stems, rising to *r* = .47 with the mechanical eligibility filter, with the strongest associations on the subscales most squarely about decentering—Meta-awareness (*r* = .49) and Disidentification (*r* = .43)—and a weaker association with Reduced Reactivity (*r* = .17); its length-controlled partial (*r* = .32, *n* = 35, *p* = .06) is filter-dependent, attenuating when unscoreable, length-only completions are retained (Supplementary Materials S4). Pooled across the two anchors (inverse-variance Fisher-*z*), the convergent correlation is *r* = .37 (95% CI [.22, .50], combined *n* = 159; script 27_convergent_pooled.py).

These magnitudes—moderate-to-large by individual-differences norms [Gignac and Szodorai, 2016]—indicate reliable but non-redundant convergence: ID taps the same construct while leaving most of its variance unshared, related but distinct rather than a substitute (Supplementary Materials S4). The observed values also understate the underlying convergence: correcting for measurement unreliability in both instruments (aggregate ID reliability .66–.75, Section 4.1; EQ-D internal consistency .83–.90 [Fresco et al., 2007]) places the disattenuated EQ-D correlation at roughly .44–.49 (Supplementary Materials S4); we report observed correlations throughout. Cross-method convergence of this kind is bounded by shared method variance and reliability. Correlations of this magnitude are typical when one construct is measured across different expression modalities (self-report versus text), much as self–informant agreement on personality has a ceiling well below unity [Connelly and Ones, 2010], and the disattenuated estimate approaches typical between-questionnaire convergence.

### 4.3 Nomological net: empathy and wise reasoning

#### Empathic concern (WASSA).

Decentering should support the compassionate, non-fused component of empathy. In 1,860 first-person essays, ID correlated with rated empathic concern at *r* = .35 (raw text); under the pre-specified eligibility filtering for this mixed-register corpus, *r* = .40 (95% CI [.36, .44], *n* = 1,705, 92% retained). The association survived adjustment for personal distress (*r* = .30) and a regression controlling distress and log length (*β* = .29, *z* = 13.0). ID thus predicts empathic concern, the compassionate (non-fused) component, over and above personal distress, as theory predicts (Supplementary Materials S5).

#### Wise reasoning about conflict (Grossmann).

If ID captures decentering rather than generic wisdom, it should predict the resolution-seeking facet of wise reasoning while leaving the adjacent subscales the theory does not implicate untouched. In 424 conflict-diary events (298 participants, subject-clustered SEs, controlling event length and survey wave), ID predicted the primary wise-reasoning subscale, Search-for-Resolution, at *β* = .18 (95% CI [.05, .30], *p* = .007), with Perspective-Taking marginal and secondary (*β* = .10, *p* = .07). The three adjacent subscales—Compromise, Recognition of Limits, and Expectation of Change—were all null (*β* = .05, .02,−.01; all *p* > .4), with 95% confidence intervals excluding standardized effects beyond |*β*| = .16: any undetected association there is at most small, consistent with discriminant specificity rather than proof of exactly zero effect (Supplementary Materials S6). We tested these coefficient differences directly with a subject-clustered bootstrap. ID’s Search-for-Resolution coefficient exceeded the mean of the three adjacent subscales by Δ*β* = .16 (95% CI [.04, .27]) and exceeded each individually (one-sided *p* ≤ .04), while remaining statistically indistinguishable from Perspective-Taking (Δ*β* = .07, n.s.), the other theory-implicated subscale. The pattern supports a decentering-specific reading rather than a generic reflectiveness factor (script 28_robustness_followups.py).

#### Adaptive reflection, not rumination.

A decentering measure should track adaptive self-examination but not repetitive negative thought, its closest discriminant neighbor. At baseline in the aging cohort, ID correlated with Rumination–Reflection Questionnaire (RRQ) self-reflection (*r* = .42), San Diego Wisdom Scale (SD-WISE) insight (*r* = .47), positive affect (*r* = .39), and overall mindfulness (Five Facet Mindfulness Questionnaire, FFMQ; *r* = .31; all *p* < .001, *n* ≈ 130), but was null with RRQ rumination (*r* = −.12, 95% CI [−.29, .05]) and negative affect (*r* = −.15, [−.31, .03]), the intervals bounding both associations below |*r*| = .31. The dissociation (reflection positive, rumination null) is what decentering predicts and not what a relabeling of verbosity or distress would produce (Supplementary Materials S11); a separate role-play corpus shows the same reduced-reactivity pattern (ECSC/COPING; Supplementary Materials S12).

### 4.4 Behavioral and incremental validity

Beyond self-report and rated text, ID was related to a behaviorally anchored outcome. In the MTurk community sample (*n* = 446), ID predicted value–behavior coherence (VBC)—the within-person alignment between stated interpersonal values and reported behavior with three close others, from separate instruments—at partial *r* = .33 (95% CI [.24, .41]; raw *r* = .37; controlling word count and English fluency; robust across eligibility-filter views, .32–.34), decomposing into normative (*r* = .27) and distinctive (*r* = .20) components. ID correlated with Agreeableness (*r* = .41) and Openness (*r* = .29), as predicted; the stricter test is whether ID predicts VBC *beyond* broad personality. Adding ID to a model with all five Big Five domains increased explained VBC variance by Δ*R*^2^ = .027 (*F*(1, 408) = 15.5, *p* = .0001; *β*_ID_ = .18), and the increment held out-of-sample (10-fold cross-validated Δ*R*^2^ = .022). ID captures value–behavior alignment that broad personality dimensions do not.

As a corroborative test, ID correlated with coherence in autobiographical narratives at raw *r* = .33, but this attenuated to partial *r* = .12 (*p* = .011) once narrative word count was controlled, indicating that shared verbal productivity carries most of the cross-task signal; we therefore treat narrative coherence as directionally consistent rather than as a primary effect (Supplementary Materials S7).

### 4.5 Discriminant validity and construct boundaries

A construct measure must also fail where the construct does not apply, and must add information beyond cheaper or more general explanations.

#### An off-construct register (CMV).

A demanding test of a measure is where it should fail: a length or articulateness proxy would win an impersonal persuasion task, but a decentering measure should not. In the ChangeMyView corpus, persuasive success is exactly such an off-construct property. ID’s paired AUC was a modest .55 (chance = .50), but this was confounded with text length (length alone gave AUC = .62). After matching arguments to within 10% on length, ID showed no positive discrimination (AUC = .45, 95% CI [.40, .49]): the apparent signal was length—the boundary the construct predicts (Supplementary Materials S8).

#### Beyond text length and verbal ability.

ID correlates modestly with verbal sophistication, as any language measure must, but the criterion associations above all survive verbosity control, and the length-matched CMV boundary and the incremental-beyond-Big-Five result are both hard to reconcile with a pure verbal-fluency account: a length/articulateness proxy would have won the (length-driven) CMV task and would not add over personality. Directly, in the neuroimaging cohort, premorbid verbal IQ (AMNART-EVIQ) correlated with ID only modestly (*r* = .33, *n* = 33), and adding it to that cohort’s criterion models shifted the ID coefficient by ≤ .02 on every outcome while AMNART itself predicted none of them (all *p* > .29; Supplementary Materials S9).

Nor is ID a demographic proxy: it related only modestly to age and sex (*r* ≈ .13) and negligibly to education (*r* = .01), and the criterion effects survived adjustment for age, sex, and education (MTurk value–behavior coherence .33 → .32; the neuroimaging stress and self-compassion effects unchanged; Supplementary Materials S3).

Within a single sample, a supervised lexical model fit on the target can match any fixed instrument—that is what supervised fitting does. The distinctive property of a measurement instrument is transfer. The fixed ID score—never trained on these labels—matched a cross-validated TF-IDF+Ridge model (a standard text classifier built from word-frequency features) fit directly on WASSA empathy (*r* = .35 vs. .36) and exceeded it on the Grossmann criterion (.20 vs. .01). Each lexical model was sample- and target-specific, and on Grossmann fell below even the length baseline when required to generalize across writers; the same single ID model remained above the length baseline on both criteria and transferred across all samples with its discriminant boundaries intact.

On the better-powered convergent test, prompting a general-purpose model did not substitute for the trained scorer. Six models prompted with the decentering definition alone (zero-shot)—ID’s own untuned base models, two mid-tier models, and the two most capable general-purpose systems available at the time of analysis (model identifiers and query dates in Supplementary Materials S15)—gave *r* ≤ .14 on EQ-D (the most capable ≤ .08), versus ID’s *r* = .31 on the matched wave (*r* = .36 grand-average). Neither enabling reasoning nor few-shot prompting with anchored exemplars (adding scored examples) moved the most capable systems off this floor (few-shot *r* = .06 and .08). The untuned base ensemble (ID’s identical architecture before tuning) scored only .07, so the convergent signal came from the contrastive fine-tuning rather than the base model or its size. The small MPoDT sample (*n* = 35) is less able to separate models, so the better-powered EQ-D is the reliable comparison (Supplementary Materials S15).

#### Beyond a human expert’s developmental score.

A close developmental benchmark for ID is a human expert’s Total Weighted Score (TWS) for the same sentence-completion protocols. As set out in Methods (Section 3.2), a substantial ID–TWS correlation is expected (from a shared, maturity-relevant axis and same-protocol scoring), so the relevant question is not its size but whether ID carries decentering-relevant information beyond it. In the neuroimaging cohort, ID correlated with TWS at *r* = .79 (95% CI [.65, .88]), sharing about 62% of its variance (*r*^2^ ≈ .62) and leaving roughly 38% unshared. We use TWS not as independent evidence for ID but as a benchmark for differential prediction. Indeed, ID predicted three decentering-relevant self-reports (the FFMQ meta-awareness and reduced-reactivity facets, and self-compassion) with little attenuation after partialling out TWS, whereas TWS tracked them only weakly (facet-level; see Supplementary Materials S9).

ID tracked self-compassion more closely than TWS (*r*_ID_ = +.49 vs. *r*_TWS_ = +.27; Williams’ *t*(39) = 2.46, *p* = .019). For quality of life the two scores diverged in sign (*r*_ID_ = +.12 vs. *r*_TWS_ = −.12; *t*(40) = 2.59, *p* = .013); since neither correlation was individually reliable, this is best read as a difference in pattern rather than a magnitude effect. Both differences held under Benjamini–Hochberg correction across the three a-priori psychological outcomes (*q* = .028). For perceived stress the two measures were directionally aligned (*r*_ID_ = −.44, *r*_TWS_ = −.33), and in joint regressions adjusting for practice group ID added more unique variance beyond TWS than the reverse for both stress (Δ*R*^2^ = .069 vs. .013) and self-compassion (.112 vs. .072); the dependent-correlation difference for stress did not reach significance in this small cohort. The ID associations also persisted controlling practice group, age, and verbosity (full test table, including the exploratory neural outcomes, in Supplementary Materials S9; a model-derived maturity score gives the same pattern, S14).

These comparisons rest on a single small cohort (*n* = 42–45 per outcome), so they are suggestive rather than decisive. Together they support the conclusion that ID shares a maturity-relevant axis with a broad expert developmental score but indexes a specific, decentering-relevant capacity not reducible to it.

### 4.6 Sensitivity to contemplative practice

#### Known-groups specificity (neuroimaging cohort).

ID distinguished practitioners from matched non-practitioner controls in the theory-predicted pattern: meditation practitioners exceeded controls at Hedges’ *g* = 1.83 (95% CI [1.08, 2.89]) and yoga practitioners at *g* = 1.38 ([0.68, 2.29]) (Figure 3B); the meditation effect was numerically larger, consistent with seated meditation more directly training decentering, though the two practitioner groups are small (*n* = 14 and 16) and the difference is not statistically distinguishable. These magnitudes reflect a cross-sectional extreme-groups comparison—long-term practitioners versus non-practitioner controls—and should not be read as expected intervention effects, which are far smaller.

Among meditators, ID exceeded *every* FFMQ facet (*g* = 1.83 vs. 1.56 next-best); for yoga practitioners ID (*g* = 1.38) was comparable to the strongest facet (Observe, *g* = 1.51) and exceeded the rest (Figure 3A). Only the two decentering-relevant facets (Observe, Non-reactivity) discriminated strongly; the three distal facets were near zero in meditators and modest in yoga (*g* ≤ .55). Within meditators (standardized to the control distribution), ID exceeded Observe (paired *t*(13) = 2.60, *p* = .022) and the three distal facets (*p* < .001) and tied the most stringent, most theory-aligned comparator, Non-reactivity (*t*(13) = 1.69, *p* = .12). The defensible claim is that ID matches the best decentering-relevant facet and exceeds all others while being a single text-derived score rather than a multi-item questionnaire—a comparison that partly reflects ID’s purpose-trained, continuous format rather than its being a better measure per se (Supplementary Materials S9).

The group separation is not an artifact of practitioners writing more: word count was non-significant alongside group in an analysis of covariance (*p* = .34), and ID’s criterion associations survive verbosity control—higher ID tracked lower perceived stress (*β* = −.34; given the a-priori directional prediction, one-sided *p* = .038, two-sided .076; controlling practice group, age, and verbosity; verbosity-controlled partial *r* = −.44, *p* = .004) and higher self-compassion (*β* = +.34, *p* = .021; verbosity-controlled partial *r* = +.46). These are cross-sectional group differences and cannot by themselves separate a practice effect from self-selection into practice; the longitudinal analysis next speaks to within-person change.

#### Practice exposure over time (aging cohort).

The aging cohort came from a randomized meditation trial, but the present secondary analysis had limited practice ascertainment and substantial exposure overlap: practice follow-up was available for only a subset, and 12 of 20 reached controls reported some meditation practice. The arm-level available-case contrast was null at 12 months (*g* = 0.02, 95% CI [−0.35, 0.40], *p* = .90; in the compliance-measured subsample, *g* = 0.27, *p* = .40) and at 24 months (*g* = −0.15, 95% CI [−0.63, 0.32], *p* = .53), with outcome availability not differing significantly across arms (Supplementary Materials S11). These estimates concern assignment in the available secondary dataset; exposure overlap would tend to attenuate an assignment effect, while missing outcome and practice data limit inference.

Analyzed instead by reported sitting-meditation exposure among those reached—a post-hoc, non-randomized analysis subject to selection on who was reached and who chose to practice—participants with any sitting practice showed greater 24-month ID change than those with none (*g* = 0.80, 95% CI [0.13, 1.56], *p* = .038; Figure 4). Sitting-meditation frequency correlated with 24-month ID change (Spearman *ρ* = .36, 95% CI [.05, .61], *p* = .017; 12-month *ρ* = .29, *p* = .042), but the continuous association attenuated after controlling baseline ID (partial *r* = .13, *p* = .40). The categorical contrast remained under baseline adjustment (adjusted mean difference = 0.65 ID points, *p* = .009; adjusted *d* ≈ 0.9; script 28_robustness_followups.py). In a joint model among reached participants, reported practice was associated with 24-month ID change (*p* = .01) whereas assigned arm was not (*p* = .50). Because exposure was not randomized and practice data were selectively available, these associations cannot distinguish an effect of practice from self-selection or other confounding. A sensitivity analysis coding never-reached participants as non-practicing preserved the categorical difference (*g* = 0.73), while the continuous dose association attenuated (Supplementary Materials S11).

## 5 Discussion

### 5.1 Main contribution

We introduced Integrated Decentering, a language-based measure that scores the decentering expressed in short reflective English text. Rather than treat it as an automated text score, we evaluated it as a psychological measurement instrument, asking whether it behaves like one across seven heterogeneous samples and multiple sources of validity evidence. The measure converged with both established decentering self-report scales (EQ-D *r* = .36; the smaller MPoDT sample corroborating); predicted the theoretically implicated outcomes—empathic concern, search-for-resolution in interpersonal conflict, and value–behavior coherence beyond broad personality—while remaining null on the adjacent constructs the theory does not implicate, including rumination and negative affect; and showed no positive discrimination in a length-matched impersonal argumentative register where the construct does not apply. It distinguished contemplative practitioners from matched non-practitioner controls (meditation *g* = 1.83), numerically more strongly for meditation than for yoga; in a post-hoc exposure analysis, 24-month gains tracked reported sitting practice; and aggregate scores were stable over 12 months. The contribution is the pattern, not any single effect. Each tested alternative account is addressed by a different cell of the design (Table 3), and no tested alternative explains the full set of results.

A second line of support is transfer: one fixed scorer, never tuned on any validation label, carried across all seven samples and matched in-domain supervised text models where they generalized and exceeded them where they did not (three samples; Supplementary Materials S15), as expected of a measurement instrument rather than a sample-specific predictor. On the well-powered convergent test it also showed convergent signal where every tested prompted model showed none—its own untuned base models and the most capable general-purpose systems available alike (matched-wave *r* = .31 vs. ≤ .14), across prompting with the definition alone, with scored examples, and with reasoning enabled (Supplementary Materials S15); the signal lies in the contrastive fine-tuning rather than the base model or its scale. This comparison should not be read as a general claim about the limits of such models under other prompting regimes; it shows only that prompting, at every capability level we tested, did not substitute for construct-targeted training on these data. To our knowledge this is the first text-derived decentering measure validated at this breadth, opening the language channel to the large-scale, multi-sample study that self-report decentering measures have long supported. For adopting researchers, transfer is the practical property: the released scorer can be applied to new samples of reflective text without retraining, within the scope conditions and usage guidance below.

Three design choices help define what ID is and is not. First, the measure is built to *fail* where the construct does not apply: on length-matched impersonal text carrying no self-relevant or emotional stake—the CMV persuasion corpus—it shows no signal beyond length, and we treat that boundary as positive evidence rather than a shortcoming. Second, the scorer is trained with a contrastive within-stage objective so that it tracks decentering expression rather than the coarse developmental *stage* it co-occurs with. Third, scoreability is treated as part of the construct: decentering is meaningful only in text containing something the writer could be fused with—a feeling, belief, value, identity, or perspective—so the eligibility filters operationalize a precondition of the construct, not a preprocessing convenience.

### 5.2 What “integrated” means

We call the measure *Integrated* Decentering because the evidence suggests that what ID captures is not merely a momentary state but the degree to which momentary decentering has shaped a person’s habitual meaning-making—a more consolidated disposition, indexed at the per-person aggregate level. Four features support this reading: (i) *stability*: ID showed year-long rank-order stability in the active-control arm not assigned meditation, despite some self-initiated practice (test–retest *r* = .69), in the range of established trait measures; (ii) *behavioral consequence*: it predicts value–behavior coherence beyond broad personality; (iii) *practice-linked change*: it is associated with sustained meditation exposure; and (iv) *developmental overlap without reduction*: it shares a maturity-relevant axis with a human expert’s developmental score (*r* = .79, as theory predicts) yet shows decentering-specific predictive structure beyond it, including decentering-relevant self-report net of that score. A measure that is stable, behaviorally consequential, sensitive to practice exposure, and maturity-relevant-but-not-reducible behaves like an index of a consolidated capacity rather than a transient state. This supports a trait-like interpretation for aggregates, while the score itself remains a property of text.

### 5.3 Relevance to mindfulness science

Contemplative research has long faced a gap between practice delivered and decentering internalized, with practice hours and trait-mindfulness self-report each capturing only part of it. ID offers a complementary, low-cost outcome. Two findings are relevant here. First, the known-groups pattern was specific: ID distinguished practitioners from controls (more so for seated meditation than for yoga), and within meditators it tied the most stringent decentering-relevant facet (Non-reactivity) while exceeding the rest. Second, the aging cohort showed a null assignment contrast alongside an association between reported practice and 24-month ID change among those with practice follow-up. Because that exposure analysis was post-hoc, non-randomized, and restricted to a selected subset, it is hypothesis-generating and does not establish that practice caused the change. It motivates preregistered testing of ID as a process or mediator measure in a trial that preserves assignment-to-exposure separation. We do not propose ID as a replacement for self-report or practice logs—each answers a question ID cannot—but as a third channel for triangulation.

### 5.4 Relevance beyond meditation

Decentering is a mechanism of broad reach, and a portable, scalable measure of it is useful wherever the capacity is a target but outcome infrastructure lags. The criterion evidence here already spans several such domains: empathic concern in response to others’ suffering (WASSA), wise reasoning about interpersonal conflict (Grossmann), and the alignment of values with action (MTurk). The same instrument is therefore relevant to psychotherapy process research—decentering is the proposed common mechanism of MBCT and the cognitive defusion of ACT—and to work on emotion regulation, conflict and negotiation, empathy, and wisdom, where reflective distance from one’s own standpoint is implicated but is hard to measure at scale. Where those traditions measure the construct by self-report—for ACT, the Cognitive Fusion Questionnaire [Gillanders et al., 2014]—ID offers a complementary text-based index that can be applied to reflective writing those literatures already collect, though it has not yet been validated against a fusion-specific scale.

A further direction concerns reflective agency in human–AI interaction: as conversational systems increasingly shape how people reason, a text-derived measure of whether someone holds an introduced interpretive frame as contestable—rather than operating through it as their own—may help index the receiver-side reflective capacity that such interactions can support or erode. We flag this as a forward direction rather than a present claim: conversational dialogue turns differ in register from the reflective writing validated here, so applications to conversational agents or digital mental health settings would require in-domain validation first; the validation here is of a decentering measure, not of any application.

### 5.5 Developmental maturity: benchmark and boundary condition

Developmental maturity is the closest benchmark for ID and a boundary condition on its neural correlates. ID correlates with an expert Total Weighted Score (TWS) at *r* = .79. This overlap is expected—a shared maturity-relevant axis that ego development and decentering are theorized to hold in common [Singleton et al., 2021], and same-protocol scoring—so we do not treat the correlation’s size as independent evidence for ID; equally, ID was not trained as a stage regressor (Section 2). Distinctness rests on differential prediction, not on a low correlation: constructs accepted as distinct routinely correlate in this range [Cronbach and Meehl, 1955, Campbell and Fiske, 1959]. Against TWS, ID tracked self-compassion more closely and added unique variance beyond it for both self-compassion and perceived stress, while diverging in sign on quality of life (Section 4.5); about 62% of ID’s variance is shared with TWS and roughly 38% is not. Within the maturity-related space, ID indexes a specific, decentering-relevant capacity rather than overall developmental level.

As a boundary condition, the exploratory neuroimaging analysis is worth reporting in brief. Computed from text alone, ID re-derived (rather than independently discovered) a developmentally gated pattern of posterior-cingulate connectivity previously reported for this cohort [Singleton et al., 2021], including a whole-sample PCC–IFC correlate (*r* ≈ .54; see S13 for *n*, confidence interval, and exact *p*) that is itself developmentally shared rather than decentering-specific. The most suggestive, and at the same time weakest, result is a stage-dependent sign inflection in the PCC–TPJ and PCC–dmPFC edges, negative below the transition to self-authoring and positive above, with the most theoretically decentering-aligned edge, PCC–TPJ, plausibly indexing the coupling of self-referential processing to perspective-taking and self–other distinction.

The caveats are heavy: it rests on an underpowered *n* = 45 split, an ID×stage interaction significant only at *p* = .046 uncorrected that does not survive multiple-comparison correction over edges and stages, and a legacy 5-minute 1.5T acquisition with inherited preprocessing. It is also a non-independent reanalysis of the very cohort whose published pattern it reproduces, in which ID overlaps the expert developmental rating at *r* = .79, so that the gating is developmental and shared with that rating rather than decentering-unique. Stage also remains confounded with contemplative practice. So bounded, the result is best read as one automated, language-derived score tracking a previously expert-gated brain pattern in the same cohort (full statistics and figure in S13), a corroborating extension, not a load-bearing pillar of the construct claim.

### 5.6 Alternative interpretations

The interpretation of ID survives the alternative accounts the data can test: each is addressed by a different part of the design, so no single confound accounts for the whole pattern (Table 3). The account most closely entwined with ID—developmental maturity—was addressed in the preceding subsection: ID is best read as a maturity-*relevant* but not maturity-*reducible* index of decentering.

### 5.7 Limitations and future directions

Several limitations bound these conclusions. *Language and register:* all validation was in English, and the scorer is validated for self-relevant, reflective or emotionally engaged text; on impersonal content with no such stake (e.g. persuasive argument about an external topic, or declarative statements) it can reduce to a length proxy, as the single length-matched CMV test indicates. *Aggregation:* the validity reported here is established at the aggregate level (per person, or per text or event for the essay and diary samples); single-stem scores should not be read on their own.

*Scorer dependence:* the specific number a passage receives depends on the model. ID has not yet been benchmarked against blinded human ratings of decentering *expression* in the same passages; convergent validity here rests on two validated self-report decentering scales (EQ-D, MPoDT), not on direct text-level human agreement (ID does predict trained-rater codings of text on the adjacent wise-reasoning constructs, but no rater judged decentering expression itself). Pending that benchmark, ID is best applied to within-study contrasts—group differences and change over time—rather than to absolute, cross-sample score levels. Scoring stability across prompt paraphrases and measurement invariance across the heterogeneous genres also remain to be established. *Sample and design:* the neuroimaging cohort was small (*n* = 45), and the aging analyses used available secondary data with incomplete long-term outcomes and practice ascertainment. ID was not a prespecified trial outcome; all aging-cohort ID analyses were post-hoc and not preregistered. The reported-practice analyses were non-randomized and restricted to participants with follow-up, so they are vulnerable to selection, self-report error, and unmeasured confounding and should not be interpreted causally. The neural analysis is likewise a non-independent reanalysis of the cohort whose published pattern it is consistent with.

#### Construct and elicitation coverage.

Within the three-process model of decentering [Bernstein et al., 2015], ID aligned most clearly with the components most directly sampled by reflective sentence completions: meta-awareness (*r* = .49) and disidentification (*r* = .43). Its association with reduced reactivity was weaker (*r* = .17). Text can express reactivity and its absence, but these sentence completions provide brief, dispositional reflections rather than in-the-moment affective challenges. Reduced reactivity may therefore be undersampled here. The estimate is imprecise (*n* = 35; approximate 95% CI [−.17, .48]), so this is a hypothesis about elicitation and construct coverage rather than a settled boundary. Future validation should test ID against state-level, behavioral, linguistic, and physiological indices of reduced reactivity. A related note concerns the scale’s low end: a low aggregate indicates little *expressed* decentering, not necessarily active fusion or distress—consistent with the rumination and negative-affect nulls—and discrimination among strongly fused registers is anchored here only by the role-play coping gradient (Supplementary Materials S12). Applications that specifically target fusion should validate that pole directly.

#### Guidance for research use.

Practical recommendations for studies adopting ID—on matching scored-text quantity to the reliability required, register eligibility, per-aggregate reporting of passage and word counts, verbosity control, and filter-view reporting—are set out in Supplementary Materials S1.1.

The corresponding agenda is clear. First and most important, scorer-independent validation against human judgement: blinded human ratings of decentering *expression* in the same passages across multiple genres, with inter-rater reliability and formal human–model agreement—the most direct check of the construct, and the top priority before operational use. Alongside it, robustness to instructed or coached “decentered-style” writing remains untested: all text analyzed here was written without knowledge of the scorer, so score inflation under deliberate impression management is an open question for elicited-text applications. Second, pre-registered replication of the practice sensitivity in a design that preserves the assignment-to-exposure contrast (e.g., a higher-barrier intervention, or contamination-monitored trials), and of the criterion associations in fresh samples. Third, independent neuroimaging with modern acquisition to test the PCC–TPJ association outside the originating cohort. Fourth, cross-cultural and non-English adaptation. Finally, application studies in the adjacent domains above—psychotherapy process, emotion regulation, conflict, and reflective agency in human–AI dialogue—where a scalable decentering measure could fill a standing gap.

### 5.8 Conclusion

Integrated Decentering is a brief, automated language measure of decentering expressed in reflective text. Across convergent, nomological, behavioral, discriminant, and practice-linked tests in seven heterogeneous samples, the pattern matched what the construct requires—with informative nulls and a clear boundary—and held beyond text length, verbal ability, and personality; with respect to developmental maturity, ID shares a maturity-relevant axis with a human expert’s score but is not reducible to it. ID can sit alongside self-report and practice-exposure measurement as a scalable internalization index for contemplative science and the many adjacent fields where decentering is a mechanism but measurement has lagged, pending the cross-scorer, cross-cultural, and pre-registered replication the evidence now motivates.

## Supplementary Material

1

Supplementary Files

This is a list of supplementary files associated with this preprint. Click to download.

- PARENTTRIALCONSORTFLOWATTRIBUTED.pdf
- CONSORT2025COMPLETED.pdf

## Figures and Tables

**Figure 1:**
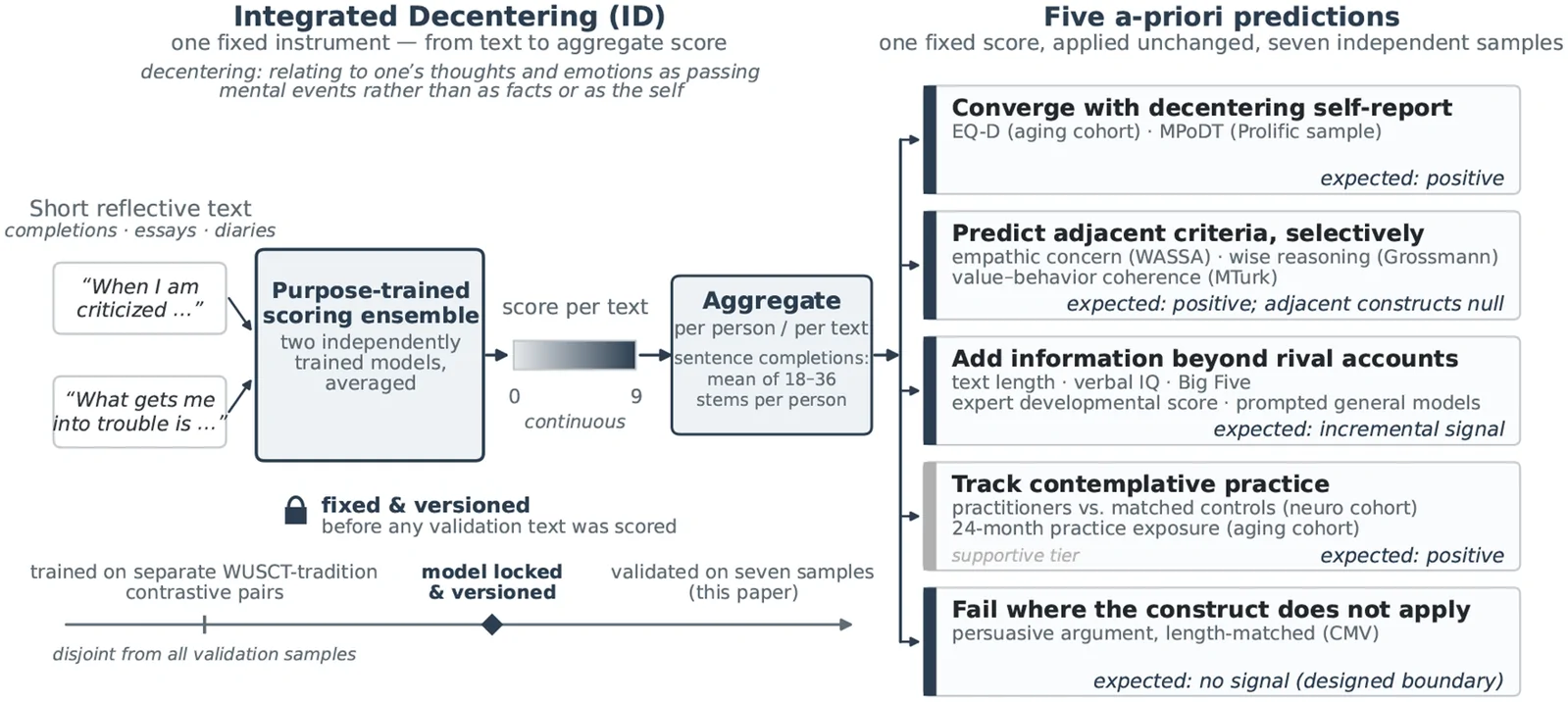
Measurement pipeline and validation blueprint for Integrated Decentering. One purpose-trained ensemble scores short reflective texts on a continuous 0–9 scale; validity is established at the aggregate level. The model was fixed before any validation text was scored and applied unchanged to all seven samples; the right panel shows the design’s five a-priori predictions, including predicted nulls and a predicted failure boundary (dark bars: core validation; grey: supportive tier). Outcomes are summarized in Table 2 and Figure 2.

**Figure 2:**
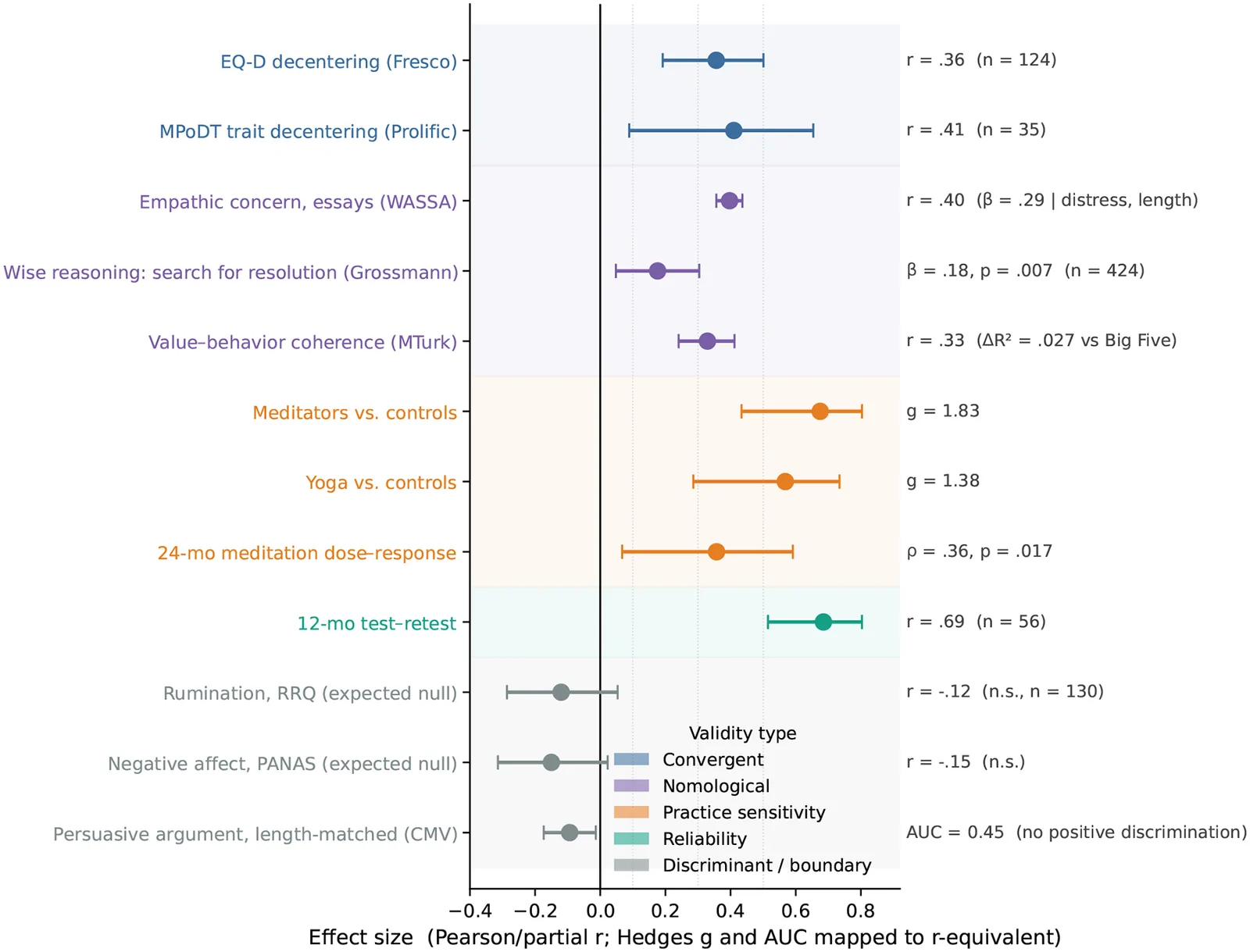
Validity evidence across seven samples for Integrated Decentering. Rows are placed on an approximate common effect-size axis for visual comparison only: Pearson/partial *r* is shown directly, while Hedges’ *g* and AUC are converted to approximate *r*-equivalents through Cohen’s *d* (AUC via the equal-variance normal approximation). Native statistics are printed at right and should be used for interpretation. Bars are 95% CIs computed from the reported *n*. The construct is supported where predicted (convergent, nomological, practice-linked), is null where it should be (rumination, negative affect), and shows no positive discrimination in the off-construct, impersonal argumentative register (CMV).

**Figure 3:**
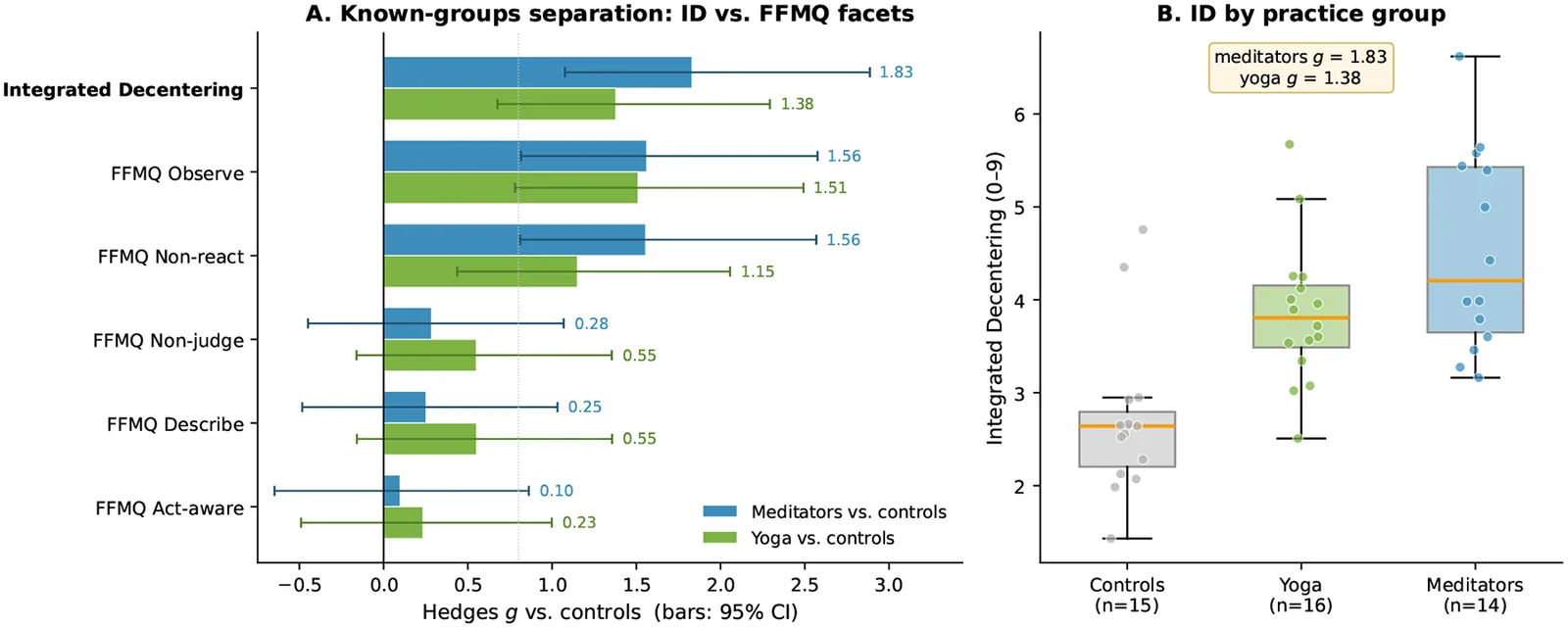
Practice known-groups separation and facet specificity (neuroimaging cohort, *N* = 45). (A) Hedges *g* vs. controls for ID and the five FFMQ facets, for meditation and yoga practitioners; ID exceeds every facet for meditators and matches the strongest facet (Observe) for yoga, with only the decentering-relevant facets (Observe, Non-react) discriminating strongly (error bars are 95% CIs). (B) ID by group: controls < yoga < meditators (*g* = 1.83 and 1.38 vs. controls).

**Figure 4:**
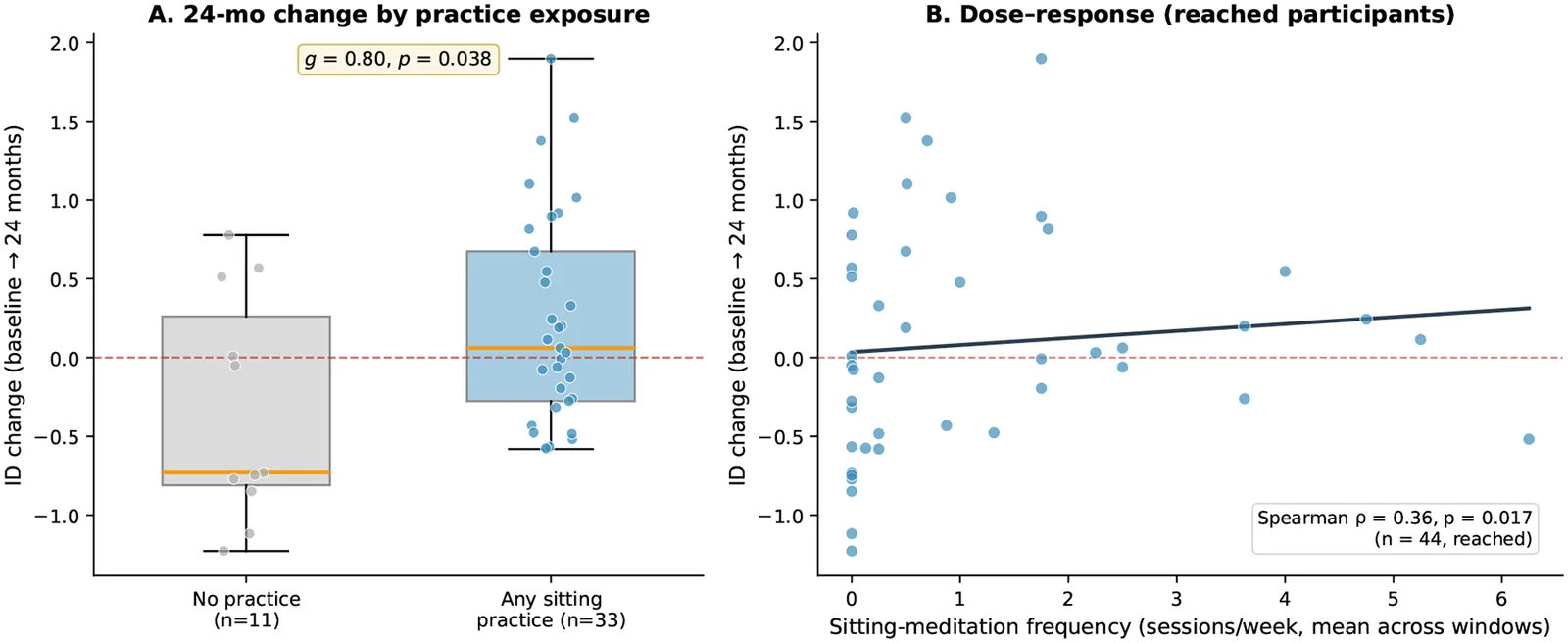
Sensitivity to contemplative practice (aging cohort). Post-hoc associations with reported sitting-meditation exposure among participants reached for practice follow-up. (A) 24-month ID change, any sitting practice vs. none (*g* = 0.80, *p* = .038). (B) Dose–response: sitting-meditation frequency vs. 24-month ID change (Spearman *ρ* = .36, *p* = .017; unadjusted—attenuates to *r* = .13 controlling baseline ID, see text). These non-randomized exposure analyses are subject to selection and confounding.

**Table 1:** Validation samples organized by the facet of construct validity they address. ID-relevant text genre and the criterion against which ID is tested are shown for each.

Validity role	Sample (*N*)	Text genre	Criterion
Convergent	MGH aging (124)	Sentence completion	EQ-D decentering [Fresco et al., 2007]
	MPoDT (35)	Sentence completion	MPoDT trait decentering [Hanley et al., 2020]
Nomological / criterion	WASSA (1,860)	Empathy essays	Empathic concern [Buechel et al., 2018]
	Grossmann (424)	Conflict diaries	Wise-reasoning subscales [Grossmann et al., 2021, Brienza et al., 2018]
	MTurk (446)	Sentence completion	Value–behavior coherence [Fournier et al., 2022]
Discriminant / boundary	CMV (4, 311 pairs)	Online argument	Persuasion, length-matched [Tan et al., 2016]
Practice-linked	MGH neuro (45)	Sentence completion	Practitioner vs. control [Gard et al., 2014, Singleton et al., 2021]
	MGH aging (143)	Sentence completion	Meditation dose; test–retest
Incremental	MTurk (446)	Sentence completion	Beyond the Big Five (BFI-2)
	MGH neuro (45)	Sentence completion	Beyond verbal IQ (AMNART); expert TWS benchmark

**Notes:** TWS = a human expert’s Total Weighted Score, a cumulative expert score of ego development for the same protocols, scored independently of the language model (see Section 3.2).

**Table 2:** Sources of validity evidence for Integrated Decentering. Headline effects by validity type; full statistics, covariates, and robustness checks are in the cited Results subsections and Supplementary Materials.

Validity type	Test	Effect	§
***Core validation***			
Convergent	EQ-D decentering (aging, *n*=124)	*r* = .36	4.2
	MPoDT trait decentering (Prolific, *n*=35)	*r* = .41	4.2
Nomological	Empathic concern (WASSA, *n*=1,705)	*r* = .40; *β* = .29	4.3
	Wise reasoning: search-for-resolution (Grossmann)	*β* = .18, *p*=.007	4.3
	adjacent wise-reasoning subscales	null (3/3)	4.3
Behavioral / incremental	Value–behavior coherence (MTurk)	*r* = .33	4.4
	beyond the Big Five	Δ*R*^2^ = .027, *p*=.0001	4.4
Discriminant / boundary	Persuasion, length-matched (CMV)	AUC = .45 (no positive discrimination)	4.5
	Rumination, negative affect (divergent)	null (*r* = −.12, −.15)	4.3
	Beyond an expert TWS score: tracks SCS more closely, QOLI sign divergence, predicts decentering-relevant self-report net of maturity	Shared axis + same protocol; not reducible	4.5
Reliability	12-mo test–retest	*r* = .69	4.1
***Supportive: practice-linked***			
Practice sensitivity	Meditators / yoga vs. controls	*g* = 1.83 / 1.38	4.6
	24-mo meditation dose–response (post-hoc)	*g* = .80; *ρ* = .36	4.6
***Exploratory extension***			
Neural	PCC–IFC (whole sample); PCC–TPJ (gated)	*r* = .54; ×stage *p*=.046	S13

**Evidence tiers:** *core* = the convergent, nomological, behavioral/incremental, discriminant, and reliability tests on which the construct claim rests; *supportive* = practice-linked evidence (cross-sectional known-groups and post-hoc exposure); *exploratory* = the non-independent neural reanalysis. Raw headline effects are shown for readability and are reported with 95% confidence intervals; secondary and covariate-adjusted estimates are reported with exact *p*-values in the cited subsections, and conclusions are unchanged under verbosity controls.

**Table 3:** Alternative accounts and the diagnostic tests that bear on each. Each is addressed by a different part of the design; no single account explains the whole pattern.

Account	Diagnostic test	Result	Verdict
Length / verbosity	Length controls; length-matched CMV boundary	No positive discrimination after matching (AUC .45 [.40, .49])	Not verbosity; do not apply to impersonal, non-self-relevant content
Verbal ability	AMNART-EVIQ adjustment	ID *β* shifts ≤ .02; AMNART n.s.	Not verbal IQ
Broad personality	Big Five incremental (cross-validated)	VBC Δ*R*^2^ = .027 (CV .022)	Not reducible to personality
Generic positivity / good reflection	Wise-reasoning subscale specificity; rumination / neg-affect nulls	Search-for-resolution +; adjacent subscales and rumination null	Selective, not generic “good reflection”
Surface linguistic distancing	LIWC-22 distancing index; incremental Δ*R*^2^	Distancing ≈ 0 with criterion in both large samples; ID adds Δ*R*^2^ = .13 (WASSA), .04 (Grossmann); distancing adds ≤ .01	Not a relabeling of surface markers
Demographics	Age, sex, education adjustment	ID × demographics ≤ .13; effects survive	Not a demographic proxy
Developmental maturity	Expert TWS benchmark; anti-shortcut training; differential-prediction tests	*r* = .79 theory-expected overlap; tracks self-compassion more closely, diverges in sign for quality of life, predicts decentering-relevant self-report net of TWS	Shared axis + same protocol; decentering-specific, not reducible
Generic LLM scoring	Zero-/few-shot prompting; base-model ablation	ID shows convergent signal where base and prompted general-purpose models do not (zero-shot, few-shot, and reasoning) on the better-powered convergent test	Signal from the trained scorer, not just prompting

## Data Availability

Several validation datasets are public: the WASSA empathic-reactions essays [Buechel et al., 2018], the ChangeMyView “winning arguments” corpus [Tan et al., 2016], and the MTurk community sample [Fournier et al., 2022] (https://osf.io/7adtj/). The Grossmann conflict-diary data are available from the original authors [Grossmann et al., 2021]. The neuroimaging cohort is a secondary analysis of [Singleton et al., 2021] and the longitudinal aging cohort a post-hoc secondary analysis of a randomized trial at the Athinoula A. Martinos Center for Biomedical Imaging [Sevinc et al., 2021] (ClinicalTrials.gov NCT02628548; registered 11 December 2015). The de-identified participant-level analytic data underlying the reported aging-cohort results, together with variable descriptions, are available from the corresponding author on reasonable request following publication, for research purposes compatible with participant consent and applicable institutional privacy requirements, and for as long as the data are retained; requests should identify the proposed analyses. The parent trial’s methods and CONSORT flow diagram are publicly available with its primary report. De-identified neuroimaging-cohort data can be requested from the authors. The MPoDT convergent sample was collected by the authors via Prolific; de-identified data are available from the authors on request. The reflective sentence-completion training material draws on WUSCT-tradition and adult-developmental corpora [Lanning et al., 2018, Madl and Cook-Greuter, 2026] that retain their own terms and are not redistributed; inquiries should be directed to the authors. The Integrated Decentering scorer (a two-adapter Qwen LoRA ensemble) is released as a gated open-weight model (access granted on request for non-commercial research use) with runnable inference and eligibility-filtering code under a CC-BY-NC-4.0 license at https://huggingface.co/tmadl/ID-decentering-scorer-ensemble. The released weights are fixed and versioned, so a result reported against ID refers to a specific, citable model version. A reproducibility package accompanies this paper containing the complete, ordered analysis code together with the reference outputs and figures it produces, so that every number, table, and figure reported here can be traced to the exact script that generated it; the build step verifies the released scorer’s documented validation correlations (MPoDT, EQ-D) exactly, confirming the pipeline. Consistent with the scorer’s gated, non-commercial, research-only release, the per-passage ID scores the scripts consume are available to researchers on request rather than posted publicly, as is the text-free scored intermediate for the supplementary ECSC/COPING check (Section S12; the underlying role-play corpus [Troiano et al., 2024] is publicly available from its authors and is not redistributed here). Analyses used Python 3.10 (NumPy, SciPy, pandas, statsmodels). The full reproducibility code (scripts, reference outputs, and figures) is available on the Open Science Framework at https://osf.io/ay532/?view_only=8250955e50124d60b3b095956a63b540.
