# Supplementary material for: Integrated Decentering: Validating an Automated Measure of Decentering Expression Across Seven Independent Samples
Source: Res Sq. 2026 Aug 14:rs.3.rs-10410851. Preprint. [Version 1] doi: 10.21203/rs.3.rs-10410851/v1 (PMC13640920; doi:10.21203/rs.3.rs-10410851/v1)
Supplement: Supplementary file 1 [file NIHPPRS10410851V1-supplement-1.pdf]

Supplementary Materials

Measuring Decentering in Reflective Language: Construct Validation of a Text-Based Scorer

All numbers below are regenerated by the accompanying reproducibility package from the per-text scorer outputs; the build step also verifies the released scorer's documented validation correlations (MPoDT, EQ-D) exactly, confirming the pipeline.

S1 Scorer architecture and training

The released scorer is an equal-weight ensemble of two independently trained LoRA adapters (Table S1). Each member scores text by *logit-expected-value decoding*: a single forward pass over the assistant scoring position reads the logits at the ten digit tokens "0"–"9", applies a softmax, and returns $\sum_{d=0}^{9} d \cdot \text{softmax}(\text{logits})[d] \in [0, 9]$. The members are strongly correlated on training-format text ($r \approx .93$) but not interchangeable across the validation battery, which motivates equal-weight averaging rather than selecting a single member or fitting validation-specific weights. Single-stem reliability is low by design—ICC$(1, 1) \approx .05$–$.09$, since one short completion carries little stable signal—which is why these single-sentence cohorts are aggregated to the per-person or per-condition level, where reliability is adequate (ICC$(1, k) = .75$ in the neuroimaging cohort, .65 in the MPoDT sample, and .66 per person-wave in the aging cohort). Because signal accrues with the quantity of scored text, reaching person-level reliability $\approx .70$ takes roughly 25–44 single sentence completions (Spearman–Brown from the single-stem ICC; `15_reliability.py`); longer first-person passages such as diary entries or essays carry more text per response and are expected to need correspondingly fewer.

Table S1: Ensemble members.

	`v9_best`	`v13_qwen36_best`
Base model	Qwen3.5-27B (4-bit QLoRA)	Qwen3.6-27B (4-bit QLoRA)
LoRA	r=32, α=64	r=16, α=32
Init	warm-start from prior SCT LoRA	fresh
Training pairs	≈62k	≈106k (broader content)
Objective	Bradley–Terry pairwise loss on E[digit softmax]	

Contrastive target and anti-shortcut training. Rather than regressing onto a developmental-stage label, members are trained on length-matched pairs to predict which passage expresses more decentering. The proxy target is a decentering-aligned indicator from upstream adult-developmental work [Madl and Cook-Greuter, 2026], treated as deliberately imperfect. Pairs are of three kinds, weighted to contribute approximately equally to the loss (Table S2). The *within-stage* pairs—same developmental stage, different decentering proxy—are the anti-shortcut signal: they force the model to track reflective distance even when stage is held constant, discouraging stage/bucket shortcut learning. They are the smallest bucket and are correspondingly up-weighted.

Training material comprised reflective sentence-completion corpora in the Loevinger/WUSCT tradition with adult-developmental extensions [Lanning et al., 2018, Madl and Cook-Greuter, 2026]. The scorer is English-only, validated for reflective text of roughly sentence-to-paragraph length, and intended for aggregate (per-person/per-condition) use, not for decisions about identifiable individuals.

Table S2: Bradley–Terry pair types and loss weights.

Pair type	Role	Weight
cross_bucket	main high-vs-low decentering contrast	0.44
within_pc	graded contrast within later adult-developmental material	1.64
within_stage	same-stage contrast (anti-shortcut)	9.43

S1.1 Scorer datasheet

We summarize the released scorer in datasheet form; architecture, training pairs, and reliability are in Section S1, and full training and inference code with versioned weights accompany the public model card.

Training target (what it is). Each member was trained to rank pairs of passages by expressed decentering using a single *decentering-aligned indicator* drawn from the upstream adult-developmental work [Madl and Cook-Greuter, 2026] as a deliberately imperfect, theory-motivated proxy. The indicator is one signal selected for its alignment with stepping back from and holding one's thoughts, affects, and identities as transient mental events; it orders training pairs by relative decentering only—it is not a regression target and not a developmental-stage or ego-level label.

What it is not. The target is not a Loevinger/WUSCT stage code, not a Total Weighted Score, and not any single Loevinger sentence-completion category; the adult-developmental corpora supply reflective text and contrastive structure, not the definition of the construct. The up-weighted within-stage pairs (Table S2) hold developmental stage constant, pushing the scorer to track reflective distance rather than maturity level; the residual overlap with an expert developmental score is reported and bounded (Sections S9, S14), and discriminant specificity rests on the large non-developmental criterion corpora (WASSA, Grossmann, MTurk), not on the small developmental cohort.

Inputs. English, first-person or self-relevant reflective text of roughly sentence-to-paragraph length, containing something the writer could be fused with (a belief, value, feeling, identity, or perspective). Purely factual or impersonal text is out of scope (main text, *Discriminant validity and construct boundaries*).

Output and score distribution. A continuous 0–9 score per passage (logit-expected-value decoding; Section S1). Across validation corpora the per-text score uses the full range, with genre-dependent location (Table S3); because absolute level varies by genre and elicitation, ID is intended for within-study contrasts (group differences, change over time), not for comparing absolute scores across samples (main text, *Measurement behavior and reliability*).

Intended use and out of scope. A research instrument for aggregate-level decentering expression. It is not validated for, and should not be used for, decisions about identifiable individuals, or to infer a person's mindfulness, wisdom, mental health, or developmental stage (main text, *The Integrated Decentering scorer*).

Guidance for research use. For studies that adopt ID we recommend: (i) matching the quantity of scored text to the reliability required—single short sentence completions are individually weak estimates of the person ($\mathrm{ICC}(1,1) \approx .05$–$.09$) and were aggregated over many stems per person here, while longer passages carry more signal per response—and reporting the reliability achieved; (ii) applying it only to self-relevant, reflective or emotionally engaged English text, not impersonal content lacking such a stake (e.g. declarative or external-topic writing); (iii) reporting the number of passages and the word counts that contribute to each aggregate; (iv) reporting which eligibility-filter view was used and confirming that conclusions hold across views; and (v) including a verbosity control (e.g., log word count) in any criterion analysis.

Table S3: Per-text ID score distribution by corpus (released v9v13 ensemble, 0–9 scale, unfiltered).

Corpus (text unit)	n	mean	SD	min	max
WASSA (essay)	1,860	3.42	2.75	0.0	9.0
Grossmann (event)	424	1.33	1.55	0.0	8.9
MPoDT (sentence)	629	3.74	3.41	0.0	9.0
MGH neuro (sentence)	1,552	3.63	3.40	0.0	9.0
ECSC role-play (reply)	1,200	2.81	2.91	0.0	9.0

S2 Eligibility filtering

Two pre-specifiable filters operationalize the precondition that a passage contain self-relevant material. The *mechanical* filter keeps a sentence if it contains a first-person pronoun, a belief/interior-state marker, or moderate affect ($|\mathrm{VADER\ compound}| \geq 0.4$); it is deterministic and local. The *LLM v2.2* filter prompts a separate annotator model to extract spans where the precondition holds (higher recall, including author-owned external-topic stances). Filter views are subset operations on the same per-text scores. For the MGH-45 stems, per-stem retention was 77.3% (mechanical), 81.0% (v2.2), and 66.6% (their conjunction). Filtering is most consequential for mixed-register text (WASSA, where the both-filters view is primary and the unfiltered view a sensitivity check), but it also removes unscoreable completions—which carry only length—from short sentence-completion data, materially de-confounding small-sample convergent estimates (Section S4).

Filter handling across analyses. We verified that no conclusion depends on the filtering choice. WASSA uses the both-filters view as primary; the MPoDT convergent estimate is reported on the scoreable subset (Section S4); MGH-45 known-groups, criterion, and ID–TWS results are stable across all/mech/v2.2/AND views (Section S14; neural in Section S13) and the group separation is not length-driven (word count $p = .34$); EQ-D is stable across views ($r = .35$–$.36$, strongest on the primary scoreable view); and MTurk value–behavior coherence is essentially unchanged across filter views (partial $r = .32$–$.34$). The Grossmann conflict-diary events are long first-person narratives that are 100% scoreable under both filters, so filtering is a no-op there ($\beta = .176$ unchanged). The MPoDT sample was the one materially affected—small n, short stems, and a non-trivial unscoreable fraction—which is why its convergent estimate is reported on the scoreable subset.

S3 Sample characteristics and demographic confounds

Demographic confounds. ID is not a demographic proxy. It correlated only modestly with age ($r = .13$ MTurk, $-.15$ neuroimaging) and sex ($r \approx .10$–$.13$), and negligibly with education ($r = .01$,

Table S4: Validation sample characteristics. Public NLP corpora carry their own (source-documented) demographics.

Sample	N	Age	% female
MPoDT (Prolific)	35	19–59	53
MGH aging (EQ-D / practice)	143	65–80	52
MTurk (Fournier)	446	M=48.4 (SD=7.3)	53
MGH neuroimaging	45	M=51.5 (SD=9.9)	59
WASSA essays	1,860	source-documented	—
Grossmann diaries	424 / 298	source-documented	—
ChangeMyView	4,311 pairs	n/a (online text)	—

MTurk). Criterion effects survived demographic adjustment: adding sex to the neuroimaging models left the self-compassion ($\beta_{\text{ID}} = +.34$) and perceived-stress ($\beta_{\text{ID}} = -.35$) coefficients unchanged (sex non-significant), and adding age, sex, and education to the MTurk value–behavior-coherence model left the partial correlation essentially intact (.33 → .32, $n = 417$). Education was unavailable for the neuroimaging and aging cohorts.

S4 Convergent validity (full)

Table S5: MPoDT sample, per-participant Pearson r ($n = 35$); EQ-D decentering, grand-average across waves.

MPoDT outcome	all stems (629)	mech-eligible (504)
Total	+.392	+.451
Meta-awareness	+.415	+.493
Disidentification	+.419	+.432
Reduced Reactivity	+.113	+.172
Composite	+.414	+.472
EQ-D decentering ($n = 124$):		
all stems	+.356	
mech-eligible	+.352	
mech ∧ LLM-scoreable	+.362	
baseline only, mech ($n = 52$)	+.417	

Verbosity robustness. The convergent associations are length-robust once unscoreable completions (which carry only length) are excluded. Both convergent anchors are reported under the same eligibility views. Controlling completion length, EQ-D is stable: partial $r = .30$ (all) → .31 (mech) → .32 (v2.2) → .33 (both), reflecting its low unscoreable fraction. For the MPoDT sample, the length-partialled association instead rises as the filters remove unscoreable completions: partial $r = .17$ (all stems, *ns*) → .28 (mechanical) → .27 (LLM v2.2) → .32 (both, $p = .06$), while the ID–length coupling falls from $r = .67$ to .57. Stripping the fixed stems (completion-only vs. full-sentence length) makes no material difference, because the stem set is common to all participants. Known-groups separation and the criterion associations are also length-robust (word count was non-significant alongside group, $p = .34$; SCS and PSS length-controlled partials +.46 and −.44; Sections S9, S15).

Disattenuation. The observed convergent correlations understate true-score convergence because both instruments are measured with error. Using the classical correction $r_c = r/\sqrt{\text{rel}_{\text{ID}} \cdot \text{rel}_{\text{EQ-D}}}$ with the aggregate ID reliabilities observed here ($\text{ICC}(1,k) = .66$ per person-wave; split-half Spearman–Brown $r = .73$; $\text{ICC}(1,k) = .75$ in the neuroimaging cohort) and published EQ-D internal consistencies ($\alpha = .83$–$.90$ [Fresco et al., 2007]), the EQ-D correlation of $r = .36$ disattenuates to approximately .44–.49 (e.g., $.36/\sqrt{.75 \times .90} = .44$; $.36/\sqrt{.66 \times .83} = .49$). The MPoDT composite ($r = .41$, $\text{ICC}(1,k) = .65$) disattenuates similarly to roughly .5. These corrected values are descriptive context, not test statistics; all inferential claims in the paper use the observed correlations.

S5 Nomological: WASSA empathic concern (full)

Theory: decentering should support empathic concern (the compassionate, non-fused component) over and above personal distress. Table S6 reports the raw-essay view (robustness) and the pre-specified eligibility-filtered views; the both-filters view is primary for this mixed-register corpus. ID is itself positively related to personal distress ($r_{\text{dist}} = .28$–$.32$ across views), as expected given that empathic concern and personal distress themselves correlate ($r = .45$); the result is therefore incremental—ID predicts empathic concern net of distress (residual-on-distress $r = .30$; $\beta = .29$ controlling distress and length)—rather than a clean dissociation.

Table S6: WASSA empathic concern by view: zero-order empathy correlation (r_{emp}), the ID–personal-distress correlation (r_{dist}), residual-on-distress r, and β (controlling distress + log length).

View	r_{emp}	r_{dist}	resid|distress	β	z	n
raw essay (robustness)	+.349	+.280	+.260	+.240	11.6	1860
mechanical	+.356	+.285	+.265	+.245	11.7	1831
LLM v2.2	+.397	+.317	+.304	+.286	13.1	1721
both (primary)	+.397	+.319	+.303	+.285	13.0	1705

S6 Nomological: Grossmann wise reasoning (full)

424 conflict-diary events (298 participants); standardized β from models with subject-clustered SEs, controlling log event length and survey wave. The primary subscale is significant; the three adjacent subscales the theory does not implicate are null, with 95% confidence intervals excluding standardized effects beyond $|\beta| = .16$ (script `20_nomological.py`).

Table S7: Grossmann wise-reasoning subscales ($n = 424$ events).

Subscale	β	95% CI	p
Search-for-Resolution (primary)	+.176	$[+.048, +.304]$	.007
Perspective-Taking (secondary)	+.101	$[-.010, +.212]$	.074
Compromise (null)	+.047	$[-.066, +.160]$	.415
Recognition of Limits (null)	+.019	$[-.064, +.102]$	.651
Expectation of Change (null)	−.009	$[-.130, +.113]$	.891

Eligibility filtering is a no-op here: the conflict-diary events are long first-person narratives, 100% scoreable under both the mechanical and v2.2 filters, so the Search-for-Resolution $\beta = .176$

is unchanged across all/mech/v2.2 views (ID–length $r = .38$, already controlled).

S7 Behavioral/incremental: MTurk (full)

ID × Big Five ($n = 418$): Agreeableness +.41, Openness +.29, Extraversion +.14, Conscientiousness +.10, Neuroticism −.10. Value–behavior coherence (controlling log word count and English fluency): overall partial $r = +.33$ (raw +.37), normative +.27, distinctive +.20 ($n = 417$–418). Incremental beyond the Big Five on VBC: base $R^2 = .263$, full $R^2 = .290$, $\Delta R^2 = +.027$, $F(1, 408) = 15.5$, $p = .0001$, $\beta_{\text{ID}} = +.18$. Narrative coherence: raw $r = +.33$, attenuating to partial $r = +.12$ ($p = .011$) controlling narrative log word count (treated as directional only).

Filter-view robustness (VBC). Value–behavior coherence is stable across eligibility views: partial r (controlling word count + English) = .33 (all) / .32 (mechanical) / .34 (v2.2) / .32 (mech ∧ v2.2), all $p < .001$ ($n = 417$). Per-sentence retention was 74% (mechanical), 74% (v2.2), 57% (both). As the filters remove unscoreable sentences the ID–length coupling falls from $r = .53$ (all) to .39 (both), yet the VBC partial is unchanged—so for this uniform first-person corpus the mechanical filter is sufficient and v2.2 adds little.

S8 Discriminant boundary: CMV (full)

For each ChangeMyView post, one comment earned a view-change award and one did not (4,311 pairs). Paired AUC for ID = .55, but text length alone gives AUC = .62: the apparent signal is length-confounded. After matching paired arguments to within 10% on length ($n = 462$ pairs), ID showed no positive discrimination (AUC = .45, 95% CI [.40, .49]); the small dip below .50 (sign test $p = .025$) is consistent with decentered, perspective-taking language being slightly less rhetorically forceful in debate, not evidence of systematic reverse prediction. Once length is controlled, ID carries no positive decentering signal in this off-construct register—the boundary the construct predicts.

S9 Neuroimaging cohort: known-groups, facets, and alternative accounts (full)

Table S8: Known-groups Hedges g vs. controls, and within-meditator paired comparison (standardized to controls).

Measure	meditators g	yoga g	within-med. ID vs. facet ($t(13)$, p)
**Integrated Decentering**	**+1.83**	**+1.38**	—
FFMQ Non-react	+1.56	+1.15	1.69, .116 (tie)
FFMQ Observe	+1.56	+1.51	2.60, .022
FFMQ Non-judge	+0.28	+0.55	5.41, < .001
FFMQ Describe	+0.25	+0.55	6.75, < .001
FFMQ Act-aware	+0.10	+0.23	7.52, < .001

Criterion regressions (ID + group + age + log word count, HC3): PSS $\beta = -.34$ ($p = .076$), SCS $\beta = +.34$ ($p = .021$), QOLI $\beta = +.23$ ($p = .28$), PCC–TPJ $\beta = +.34$ ($p = .27$).

Table S9: ID compared with human-coded TWS by outcome ($r_{\text{ID,TWS}} = .79$; per-outcome $n =$ 42–45 after listwise deletion). TWS is an independently scored, cumulative expert score of ego development for the same sentence-completion protocols, with which ID shares a maturity-relevant axis (and same-protocol text). Williams' t for two dependent correlations sharing a variable ($df = n - 3$) tests whether ID and TWS differ in how closely they track each outcome; p is two-tailed. The three psychological outcomes (PSS, QOLI, SCS) form the a-priori family; under Benjamini–Hochberg correction within that family SCS and QOLI hold at $q = .028$ (treating all six tests as one family gives $q = .056$). The three neural rows are exploratory and uncorrected. ΔR^2 partitions unique contributions in joint regressions adjusting for practice group. A model-derived maturity-score robustness check gives the same pattern (SCS $t = +2.71$, QOLI $+2.71$, PSS -1.12; Section S14). The PCC–TPJ edge is available for 43 of 45 participants (TPJ ROI coverage missing for two); PCC–IFC and PCC–dmPFC are complete ($n = 45$).

Outcome	n	r_{ID}	r_{TWS}	Williams' t	p	$\Delta R^2_{\text{ID}\mid\text{TWS}}$	$\Delta R^2_{\text{TWS}\mid\text{ID}}$
PSS	43	−.44	−.33	−1.20	.236	.069	.013
QOLI	43	+.12	−.12	+2.59	.013	.085	.098
SCS	42	+.49	+.27	+2.46	.019	.112	.072
PCC–TPJ	43	+.24	+.17	+0.72	.473	.051	.000
PCC–IFC	45	+.54	+.52	+0.24	.809	.042	.014
PCC–dmPFC	45	+.20	+.24	−0.47	.641	.000	.034

Less stage-bound than the expert TWS score. ID is developmentally graded without reducing to discrete stage. A clarification first: TWS is a *cumulative* maturity score summed across responses, whereas developmental stage is assigned by applying threshold (cut-off) rules to the protocol; because the same total can arise from different response profiles and adjacent stages span overlapping score ranges, the score-to-stage map is many-to-one. A high correlation with TWS is therefore uninformative about whether ID recovers discrete stage; even ID = TWS would not make ID = stage. In the neuroimaging cohort, which spanned conventional through postconventional developmental levels (stages 3–5, ∼16% postconventional), ID correlated with human-coded developmental stage at $r = .69$ (Spearman $\rho = .64$, $n = 45$)—well below the $r = .95$ for TWS. Squaring these, discrete stage accounts for about 48% of ID's variance against roughly 90% of TWS's—roughly half—so the within-stage contrastive training attenuated, rather than eliminated, the stage association. ID nonetheless tracked TWS within stage (partial $r(\text{ID}, \text{TWS} \mid \text{stage}) = .63$): it indexes continuous reflective distance more than the stage label, while retaining the shared-axis correlation theory expects.

Decentering-relevant self-report net of developmental maturity. Within the neuroimaging cohort ($n = 43$ with complete self-report), ID predicted three decentering-relevant self-reports, and these associations were largely retained after controlling expert developmental maturity. ID correlated with the FFMQ Observe facet (a meta-awareness index) at $r = +.50$; the partial controlling TWS was $+.49$ ($p = .001$) and controlling discrete stage $+.48$ ($p = .001$), whereas TWS itself correlated with Observe only at $+.27$. For the FFMQ Non-reactivity facet (reduced reactivity), $r = +.50$; partial controlling TWS $+.41$ ($p = .006$) and controlling stage $+.52$ ($p < .001$), versus $+.33$ for TWS. For self-compassion (SCS), $r = +.49$; partial controlling TWS $+.47$ ($p = .002$) and controlling stage $+.54$ ($p < .001$), versus $+.27$ for TWS. Observe and Non-reactivity map onto two of the three decentering components in the Bernstein–Fresco model (meta-awareness and reduced reactivity) [Bernstein et al., 2015]. ID's convergence with decentering-relevant self-report is thus

largely retained when developmental maturity is partialled out (Observe and self-compassion essentially unchanged; Non-reactivity attenuated modestly, .50 to .41), while maturity itself tracks these facets only weakly. Two caveats temper this: the indices are mindfulness *facets* rather than full decentering scales (e.g., the EQ-D [Fresco et al., 2007] or MPoDT), which were not co-measured with TWS in any cohort, and the estimates come from a single small cohort ($n = 43$). The pattern is therefore suggestive rather than decisive.

Beyond verbal IQ. ID correlated modestly with premorbid verbal IQ (AMNART-EVIQ; $r = .33$, $n = 33$), as expected for a language measure. Adding AMNART-EVIQ to each core criterion model (outcome $\sim$ ID + group + age + log word count) shifted the ID coefficient negligibly—$\Delta\beta_{\text{ID}} = -.010$ (PSS), +.010 (QOLI), $-.015$ (SCS), $-.000$ (PCC–TPJ), $-.001$ (PCC–IFC)—and AMNART itself predicted none of the outcomes (all $p > .29$). Consistent with the length-matched CMV boundary (Section S8) and the incremental-beyond-Big-Five result (Section S7), the criterion associations are not attributable to verbal fluency.

S10 Parent aging trial and secondary-analysis reporting

Registration and design. The parent study, "Neural and Cognitive Changes Associated With Mental Training in Older Adults," was a single-site, two-arm, parallel randomized trial at Massachusetts General Hospital, Boston. It was registered at ClinicalTrials.gov on 11 December 2015 (NCT02628548) and ran from 18 January 2016 to 31 December 2021. The registry records 175 participants enrolled in the overall study. The primary trial report [Sevinc et al., 2021] records 1,472 screened and 146 randomized 1:1 to mindfulness training ($n = 72$) or cognitive-fitness training ($n = 74$). The discrepancy is one of study stage: registry enrollment, randomization in the primary report, and availability in this secondary analysis are distinct denominators. Full parent-trial methods are publicly available in the published report, with its published CONSORT flow diagram provided as supplementary material.

Participants, interventions, randomization, and masking. The parent trial recruited cognitively normal, meditation-naive, right-handed English-speaking adults aged 65–80 through advertising, direct mail, and email lists; full medical, neuropsychiatric, MRI, cognitive, medication, and prior mind–body-practice criteria are reported by Sevinc et al. [Sevinc et al., 2021]. After baseline testing, the REDCap randomization module assigned participants 1:1 in permuted blocks of six stratified by gender; the allocation sequence was generated and concealed within this centralized module, so the personnel who enrolled participants and assigned them to the interventions could not view the sequence or upcoming allocations—each assignment was revealed by the module only at the point of randomization, after enrollment. The registry classifies masking as triple (participants, investigators, and outcome assessors); the primary report specifies that testing staff were blinded to assignment and that participants were told both programs promoted cognition to reduce expectation differences. The mindfulness program comprised eight weekly one-hour classes (45 minutes of practice plus 15 minutes of check-in/instruction) and 45 minutes of daily home practice; the active cognitive-fitness comparator matched class and home-practice time using puzzles. No concomitant care was delivered in either arm: beyond the assigned program, the trial provided no additional treatment or care. Mean attendance was 7.05 versus 6.64 of eight classes and mean home practice 4.01 versus 5.99 hours/week, respectively. Instructor qualifications and complete intervention content are reported in the primary report [Sevinc et al., 2021].

Outcomes and analysis status. The registered parent-trial primary outcomes were change in brain structure and brain activity at eight weeks; registered secondary outcomes included biomarkers and cognition. ID was not a registered or prespecified outcome. Every ID analysis in this paper is a post-hoc secondary analysis and was not preregistered. No sample-size calculation, interim analysis, stopping rule, or harms analysis was conducted for ID. ID analyses used all available observations meeting the requirements of each stated analysis, without imputation; exact statistical methods are described in Section 3.2. The parent trial completed follow-up as planned, and this report makes no claim about intervention benefits or harms.

Secondary-analysis flow. The published parent-trial CONSORT diagram reports that 67 of 72 mindfulness participants and 71 of 74 cognitive-fitness participants began treatment; five participants per arm discontinued treatment, one per arm was lost to follow-up, and 61 and 65, respectively, were analyzed for the parent trial's cognitive outcome, with reasons reported in the diagram [Sevinc et al., 2021]. Sentence-completion text was available for 143 participants at one or more analyzed waves: 142 at baseline, 119 at 12 months, and 78 at 24 months. In the available secondary export, 120 participants were linked to one of the two randomized arms (58 mindfulness, 62 cognitive fitness); 23 records without an eligible two-arm link were excluded from arm comparisons. Complete baseline-to-12-month change was available for 53 mindfulness and 56 control participants; complete baseline-to-24-month change for 38 and 32, respectively. Reasons for missing long-term sentence-completion or practice data were not available in the secondary export. The 12-month arm contrast was $g = 0.02$ (95% CI $[-0.35, 0.40]$; mindfulness change $M = -0.10$, SD $= 0.68$; control $M = -0.12$, SD $= 0.71$); at 24 months it was $g = -0.15$ (95% CI $[-0.63, 0.32]$; mindfulness $M = -0.07$, SD $= 0.69$; control $M = 0.04$, SD $= 0.68$). Published parent-trial baseline characteristics and its original outcomes are reported in Tables 1–2 of Sevinc et al. [Sevinc et al., 2021]; the present report does not re-report those prespecified outcomes.

Table S10: Parent-trial and present secondary-analysis participant accounting. Dashes indicate that the public aggregate was not reported by arm.

Stage	Total	Mindfulness	Cognitive fitness
Registry enrollment	175	–	–
Randomized in the primary report	146	72	74
Begun treatment in the primary report	138	67	71
Parent cognitive outcome analyzed	126	61	65
Linked in the present secondary export	120	58	62
Complete baseline-to-12-month change	109	53	56
Complete baseline-to-24-month change	70	38	32

Table S11: Published parent-trial baseline characteristics (available cases), reproduced numerically from Table 1 of Sevinc et al. [Sevinc et al., 2021]. These describe the parent trial, not the present secondary analytic sample.

Characteristic	Mindfulness	Cognitive fitness
Age, years	70.2 ± 4.1 ($n = 70$)	71.0 ± 4.3 ($n = 75$)
Female	55.7% ($n = 70$)	53.3% ($n = 75$)
Education, years	16.7 ± 1.8 ($n = 69$)	16.7 ± 1.9 ($n = 74$)
Education, ISCED level	6.5 ± 1.0 ($n = 69$)	6.5 ± 1.1 ($n = 74$)

S11 Aging cohort: practice sensitivity and stability (full)

Practice follow-up was available for a subset, and exposure overlapped across arms: among reached participants, the mindfulness arm's sitting-practice composite was 1.31 versus 0.35 in controls, while 12 of 20 reached controls reported some practice (Fisher $p = .08$ for any practice by arm). The available-case arm contrast was null at 12 months ($g = 0.02$, $p = .90$) and 24 months ($g = -0.15$, $p = .53$); outcome availability did not differ significantly by arm at 24 months (66% vs. 52%, $p = .14$). In a post-hoc joint model among 36 reached participants with 24-month change, reported practice was associated with change ($b = +0.70$, $p = .011$) whereas assigned arm was not ($p = .50$). This non-randomized exposure association is subject to selection and confounding. Exposure-based analyses and sensitivity checks are reported in Table S12; test–retest in the active-control arm was $r = .69$ ($n = 56$, 12 months), comparable at 24 months.

Table S12: Meditation dose–response and categorical contrasts (reached participants), new scorer.

Test	12 months	24 months
Dose, baseline-adj. partial r	+.26 ($p = .08$)	+.13 ($p = .40$)
Dose, Spearman ρ	+.29 ($p = .042$)	+.36 ($p = .017$)
Any practice vs. none, g	+.29 ($p = .35$)	+.80 ($p = .038$)
n (reached)	48	44

Baseline nomological net (discriminant dissociation). At baseline, ID (scoreable view, grand-average) tracks adaptive self-examination and wellbeing but not repetitive negative thought—the key discriminant test for a decentering measure (Table S13). Reflection, insight, and positive affect correlate positively; RRQ rumination and negative affect are null. (The EQ-D rumination subscale is weakly positive, $r = .23$, distinct from the RRQ repetitive-negative-thought scale.) This adaptive-versus-ruminative split is not what a relabeling of verbosity, distress, or general maturity would yield.

Table S13: Aging cohort baseline: ID vs. adaptive self-examination and repetitive negative thought. RRQ, Rumination–Reflection Questionnaire; PANAS, Positive and Negative Affect Schedule.

Criterion	r (95% CI)	n
RRQ self-reflection (adaptive)	+.42 [.27, .55]	133
SD-WISE insight	+.47 [.32, .60]	125
PANAS positive affect	+.39 [.23, .52]	133
FFMQ total mindfulness	+.31 [.15, .46]	132
PANAS negative affect	−.15 (*ns*)	133
RRQ rumination (RNT)	−.12 (*ns*)	133

S12 Reduced reactivity in coping responses (ECSC/COPING)

As an exploratory check on the reduced-reactivity facet of decentering, we scored 1,200 role-play responses from the Emotion and Coping Strategy Corpus (ECSC/COPING) [Troiano et al., 2024], in which a writer replies to a provocative statement under one of four assigned coping strategies—attack, reject, distance, contact—across five charged topics (reproducible scripts `08_ecsc_prep.py`–

10_ecsc_analyze.py). ID followed the predicted reactivity gradient, lowest for the reactive strategies and highest for engaged contact (attack 2.0 $<$ reject 2.3 $<$ distance 2.7 $<$ contact 4.3). The reactive (attack/reject) versus non-reactive (distance/contact) contrast was substantial ($g = -0.47$) and held controlling response length and topic (partial $r = -0.24$, $p < .001$); a length-only classifier did not separate the strategies in the predicted direction (AUC 0.34, at or below chance), while adding ID raised it to 0.56, and a within-reactive placebo (attack vs. reject) was null—so the signal is the reactive stance, not verbosity. Within the corpus's per-response emotion ratings, ID was selectively negative for reactive, aversive emotions (anger, disgust, contempt, dislike; partial $r \approx -.18$, net of length) yet positive for sadness (+.11) and null for guilt, shame, and regret, tracking a non-reactive stance rather than negative affect in general; the contrast persisted even when rated hostile-emotion content was partialled out (partial $r = -.17$), a conservative check since reduced reactivity is itself part of decentering. These role-play data are exploratory and correlational, but the pattern is the one a reduced-reactivity component of decentering predicts.

S13 Neuroimaging: connectivity detail and developmental gating (full)

As an exploratory extension we asked whether ID—derived purely from text—recovers a brain signature previously reported for this cohort. Singleton et al. [Singleton et al., 2021] analyzed the same 45 participants with an expert-coded developmental rating and the same PCC-seed resting-state connectivity, reporting (i) PCC–inferior-frontal-cortex (IFC) coupling as the primary whole-sample correlate and (ii) PCC–temporoparietal-junction (TPJ) and PCC–dorsomedial-prefrontal (dmPFC) coupling *sign-inflecting* at a developmental boundary (the transition to self-authoring), positive above and negative below. Because that boundary and those regions are fixed by prior work on this exact cohort, they provide a principled, hypothesis-constrained test not chosen here. However, because we reanalyze the same data and ID is highly correlated with that study's developmental rating, it is a non-independent consistency check, not independent confirmation.

ID showed the same pattern from text (Figure S1). PCC–IFC, the whole-sample edge, correlated with ID at $r = .54$ ($\beta = .49$, $p = .009$, controlling group), robust across all eligibility-filter views ($\beta = .44$–$.50$, all $p < .012$). PCC–TPJ was *developmentally gated*: at or below the boundary the ID–TPJ association was slightly negative ($r = -.22$), above it clearly positive ($r = +.50$), with an ID$\times$stage interaction at $p = .046$ (uncorrected across the three edges, not surviving multiple-comparison correction; leave-one-out stable in sign), and the full-sample association correspondingly diluted ($\beta = .40$, $p = .14$)—the same pre/post averaging Singleton's results imply. PCC–dmPFC showed the same gated pattern (interaction $p = .010$). The expert developmental rating reproduced the identical gating on the same edges (TPJ and dmPFC interactions both significant; IFC whole-sample), confirming that our connectivity measures are the regions Singleton reported and that the gating is a general developmental phenomenon shared by ID and the expert score.

We frame this as exploratory convergent evidence, not as an incremental claim. The gating is developmental and shared with the expert rating, so it is not decentering-unique; the cohort is small; developmental stage and practice are confounded here as in the original report; the TPJ variance partition into ID-specific versus shared components is unstable under leave-one-out, so we claim no ID-specificity beyond maturity at this edge; and connectivity was computed from the original 5-minute 1.5 T acquisition with the preprocessing of [Singleton et al., 2021], inherited without an independent motion or physiological-noise reanalysis.

The expert TWS rating shows the same gating on the same edges, confirming that the connectivity measures are the regions reported by Singleton et al. [Singleton et al., 2021] and that the

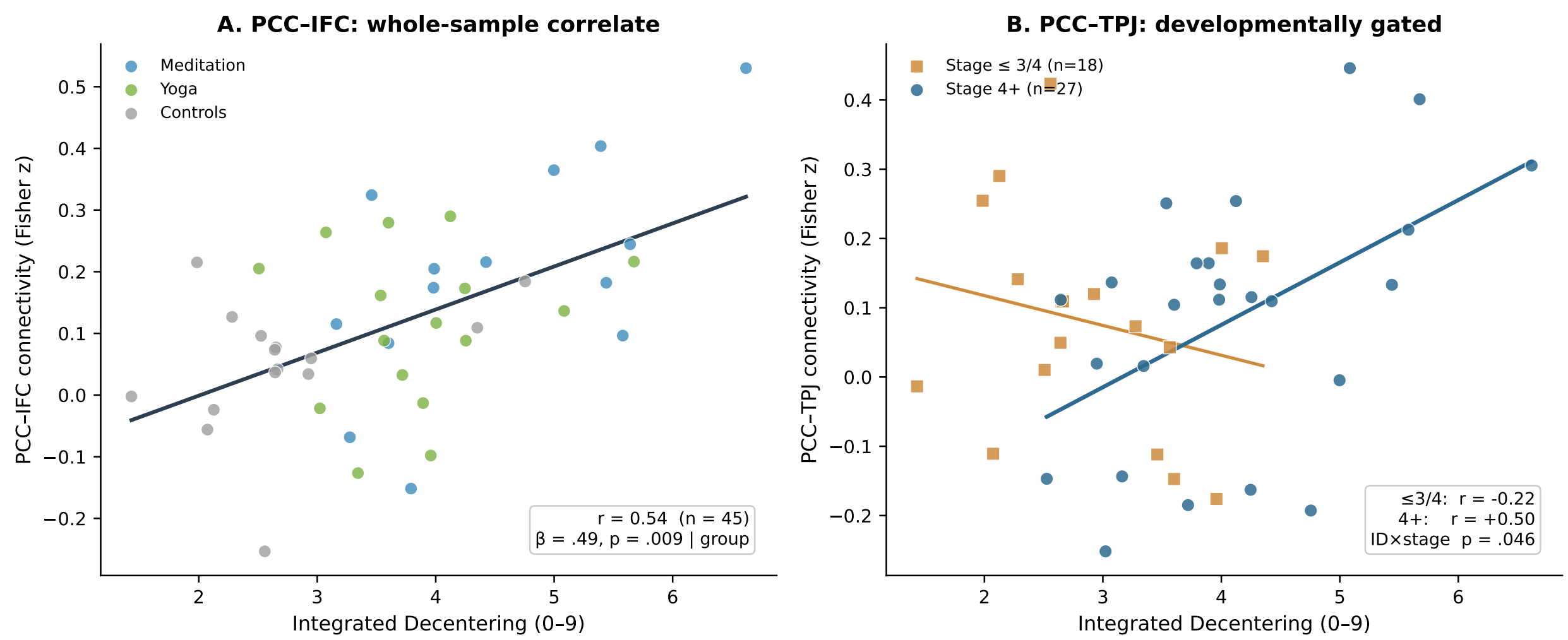

Figure S1: **Exploratory: ID recovers a developmentally gated PCC-connectivity pattern (neuroimaging cohort).** (A) PCC–IFC connectivity vs. ID, whole sample ($r = .54$; $\beta = .49$, $p = .009$ controlling group), coloured by practice group. (B) PCC–TPJ connectivity vs. ID, split at the human-coded developmental boundary (Stage $\leq 3/4$ vs. 4+): the association is negative below and positive above (ID$\times$stage $p = .046$), echoing Singleton et al. [Singleton et al., 2021]. Exploratory; small subgroups; see text for limits.

Table S14: PCC-seed connectivity by eligibility-filter view (group-controlled β, HC3).

Edge	all	mech	v2.2	both
PCC–IFC	.50**	.50**	.47**	.44*
PCC–TPJ	.40	.35	.41	.35
PCC–dmPFC	.20	.17	.25	.20

$^{*}p < .05$, $^{**}p < .01$. PCC–IFC robust across views; PCC–TPJ/dmPFC not significant whole-sample (see gating below).

inflection is a general developmental phenomenon (shared by ID and TWS), not decentering-unique. A group-based contrast (control vs. practitioner) gives the same picture, as expected since stage and practice are confounded in this cohort (controls $\approx\leq 3/4$; practitioners ≥ 4). Within the post-threshold subgroup, the partition of PCC–TPJ variance into ID-specific versus shared components is unstable under leave-one-out, so we do not claim ID-specificity beyond maturity at this edge. Range restriction is present (post-threshold ID $M = 4.2$, SD$= 1.1$; pre $M = 2.9$, SD$= 0.8$).

S14 Robustness checks

Model-derived maturity (robustness for Section S9). Repeating the ID–TWS comparison with a model-derived maturity score in place of the human rating gave the same pattern: self-compassion and the quality-of-life sign divergence were significant (Williams' $t = +2.71$ for both), while perceived stress was directionally consistent but not significant ($t = -1.12$). The human-coded rating is method-independent of the language model and is therefore reported in the main text.

Table S15: Developmental-stage moderation (human stage; pre $\leq 3/4$, $n = 18$; post 4+, $n = 27$). Subgroup r and ID×stage interaction p, for ID and for the expert TWS rating.

	ID			TWS (expert)		
Edge	r_{pre}	r_{post}	int. p	r_{pre}	r_{post}	int. p
PCC–IFC	+.18	+.62	.24	+.02	+.64	.31
PCC–TPJ	−.22	+.50	.046	−.26	+.39	.025
PCC–dmPFC	−.35	+.41	.010	−.47	+.45	.003

Filter-view robustness. Known-groups separation, the criterion regressions, and the ID–TWS correlation are stable across eligibility-filter views (e.g., meditator $g = 1.54$–1.83; $r_{\text{ID,TWS}} = .79$–$.81$ across views), confirming that none of the conclusions depends on a particular filtering choice.

Verbal IQ. The AMNART-adjusted criterion reanalysis is reported in Section S9: adding premorbid verbal IQ leaves the ID coefficients essentially unchanged and AMNART predicts none of the criteria, so verbal fluency does not carry the ID–criterion signal.

S15 Baseline comparison

ID is a *fixed* scorer with no parameters fit on these labels. We compare its simple correlation with each criterion against (i) a length-only baseline (log word count) and (ii) a TF-IDF + Ridge model trained directly on the criterion and evaluated out-of-sample (5-fold cross-validated predictions; GroupKFold by writer for the repeated-measures Grossmann corpus). The supervised baseline has every advantage—it is fit on the exact target in the exact corpus—yet ID matches it where it generalizes (WASSA), exceeds it where it does not (Grossmann, across writers, where the supervised model drops below the length baseline), and ID exceeds length on both nomological criteria, the supervised model only on WASSA (Table S18). The *same* ID scorer transfers across all three corpora, whereas each TF-IDF model is corpus- and target-specific. The one place a length baseline matches ID is the small MPoDT convergent sample (all stems); this reflects unscoreable completions that carry only length—on the scoreable subset ID exceeds length and predicts MPoDT beyond it (Section S4). In Table S18, r_{ID} is the zero-order correlation; the length- and survey-wave-controlled Grossmann estimate is $\beta = .18$ (Section S6).

Surface linguistic-distancing baseline. Automated work on the neighbouring construct of psychological self-distancing indexes it through surface lexical markers—low first-person-singular pronoun use, more articles, more analytic/abstract language [Nook et al., 2017, Kross and Ayduk, 2017]. We tested whether ID merely recovers such markers by scoring every criterion text with the validated LIWC-22 dictionary [Boyd et al., 2022] (reproducible scripts `26a_make_liwc_csv.py` and `26b_liwc_distancing.py`) and forming (i) a Nook-style distancing composite (equal-weight z-scores: −first-person-singular, +articles, +past- vs. −present-focus, +big words, +analytic thinking, +non-first-person reference, +cognitive processing), (ii) LIWC's analytic-thinking summary index alone [Pennebaker et al., 2014], and (iii) the best out-of-sample linear combination of the full distancing-relevant category set (5-fold cross-validated Ridge; GroupKFold by writer for Grossmann). In the two well-powered corpora the distancing index is essentially unrelated to the criterion (WASSA empathic concern: composite $r = .03$, Analytic .07, supervised-CV .16, vs. ID .35; Grossmann search-for-resolution: composite −.07, Analytic −.03, supervised-CV −.02, vs. ID .20), so

ID's incremental contribution is large while the distancing index adds almost nothing (WASSA $\Delta R^2_{\mathrm{ID|dist}} = .13$ vs. $\Delta R^2_{\mathrm{dist|ID}} = .01$; Grossmann .04 vs. .00, ID $p = .001$ cluster-robust by writer; Table S16). On the small MPoDT convergent sample ($n = 35$) the distancing index does track the criterion ($r = .38$) and correlates with ID ($r = .55$)—unsurprising where the criterion is itself a self-report decentering scale—but $n = 35$ cannot partition unique variance (the supervised combination is not estimable at this size), so the discriminant evidence rests on the two large corpora. ID is not a relabeling of surface distancing markers.

Table S16: Surface linguistic-distancing baseline (LIWC-22) vs. ID. r_{dist} is the equal-weight Nook-style distancing composite; r_{Analytic} is LIWC's analytic-thinking summary index alone [Pennebaker et al., 2014]. ΔR^2 partitions unique variance in the joint model (ID + composite). In the two large corpora the distancing index is near zero with the criterion and adds almost nothing beyond ID; at MPoDT ($n = 35$) the index tracks the criterion but the sample is too small to partition unique variance (increments not significant).

Corpus / criterion	n	r_{ID}	r_{dist}	r_{Analytic}	$\Delta R^2_{\mathrm{ID\|dist}}$	$\Delta R^2_{\mathrm{dist\|ID}}$
WASSA: empathic concern	1860	+.35	+.03	+.07	+.13	+.01
Grossmann: search-for-resolution	424	+.20	−.07	−.03	+.04	+.00
MPoDT composite	35	+.41	+.38	+.28	+.06	+.03

Zero-shot LLM baselines. A second baseline asks whether the contrastive fine-tuning adds anything over simply prompting a capable model. We zero-shot six models with a strong decentering rubric (temperature 0, reasoning disabled, per-completion scoring identical to ID's pipeline; reproducible script `19_zeroshot.py`): the *same* two base models ID fine-tunes (Qwen3.5-27B + Qwen3.6-27B; their average is the untuned base ensemble—a direct ablation), two mid-tier models (GPT-4.1-mini, Claude-3.5-Haiku), and the current frontier models (GPT-5.5, Claude Opus 4.8). All comparisons are matched: every model is scored on the same stems and aggregated identically, on both the all-stems and scoreable (mech $\wedge$ v2.2) views. On the well-powered EQ-D ($n = 124$), ID substantially exceeds the zero-shot models on either view (ID .31 all / .33 scoreable): the untuned base ensemble (.07/.08), the mid-tier models ($\leq .14$), and the current frontier models, which do no better and approach zero (GPT-5.5 .08/.01, Claude Opus 4.8 .02/−.01). Across the zero-shot, few-shot, and reasoning-enabled prompting tested, capability and scale did not substitute for the targeted fine-tuning. On the small MPoDT sample ($n = 35$) estimates are noisy and not distinguishable (GPT-5.5 .48/.46 brackets ID's .41/.48; Opus 4.8 is low at .21/.23), so the powered EQ-D is the reliable comparison. Beyond accuracy, zero-shot frontier scoring is non-reproducible (model versions drift) and uncalibrated, whereas ID is a small, fixed, deterministic, released instrument. All prompted models were queried through the OpenRouter API at temperature 0 with reasoning disabled (the reasoning-enabled check used reasoning effort "low"), one completion per call; the system prompt and scoring rubric are in the reproducibility scripts (`19_zeroshot.py`, `19d_fewshot.py`, `19c_zeroshot_or_reasoning.py`). Because prompted-model outputs drift with provider updates, we pin the model identifiers and query dates: the zero-shot EQ-D comparison used `qwen/qwen3.5-27b`, `qwen/qwen3.6-27b`, `openai/gpt-4.1-mini`, `anthropic/claude-3.5-haiku`, `anthropic/claude-opus-4.8`, and `openai/gpt-5.5` (queried 2026-05-29); the reasoning-enabled runs of the two most capable models were made on 2026-06-12 and the few-shot runs on 2026-07-04. Per-completion scored outputs for every model are retained in the reproducibility package.

Pretraining exposure. Several validation corpora (WASSA, CMV, the MTurk sample) are public and may occur in the pretraining data of the Qwen base models; the private MGH cohorts and the author-collected MPoDT sample cannot. Three features of the design bound this concern. First, the scoring task is judging a passage presented in isolation, not recalling it: no criterion label accompanies any text at inference, and the public releases pair labels with texts only as research-download metadata. Second, the untuned base ensemble—identical pretraining, no contrastive fine-tuning—shows no convergent signal ($r = .07$), so pretraining exposure alone demonstrably does not produce the validation pattern; the signal enters with fine-tuning, whose material is non-public and disjoint from every validation sample (Section S1). Third, the convergent (EQ-D), reliability, and practice-linked results all come from the private clinical cohorts. Memorization is therefore an implausible account of the pattern.

Few-shot frontier check. Because zero-shot prompting might understate what in-context examples can elicit, we re-ran the two frontier models on the identical EQ-D pipeline with the same rubric plus six author-written anchor exemplars spanning the 0–9 scale, presented as in-context dialogue turns (scoring-manual-style anchors; synthetic, so no training or validation text enters the prompt; temperature 0, reasoning disabled; reproducible script `19d_fewshot.py`). Few-shot prompting did not produce convergent signal: Claude Opus 4.8 $r = .06$ (zero-shot .02) and GPT-5.5 $r = .08$ (zero-shot .08), against ID's $r = .31$ on the matched wave. We did not tune the exemplar set; as with the other prompted baselines, some untested prompt could conceivably do better, but rubric, exemplars, and reasoning each failed to move the frontier models off floor on this test.

Reasoning-enabled frontier check. Because the two frontier models are reasoning-first systems, we re-ran them with reasoning enabled (pinned OpenRouter API, reasoning effort low, one completion per call, 3,042 scoreable EQ-D completions across 123 persons; reproducible script `19c_zeroshot_or_reasoning.py`). On the identical scoreable view, reasoning did not produce convergent signal: GPT-5.5 $r \approx .00$ ($p = .99$) and Claude Opus 4.8 $r = .03$ ($p = .72$), against ID's $r = .33$ on the same view (the reasoning-disabled estimates on this view are .01 and $-.01$). This comparison was run on the powered EQ-D test only; the small MPoDT sample ($n = 35$) cannot separate scorers at any operating point, as the reasoning-disabled estimates already show.

Table S17: Zero-shot LLM decentering scoring vs. ID, per-person, on the convergent criteria. Models span the open base (Qwen3.5/3.6-27B), mid-tier (GPT-4.1-mini, Claude-3.5-Haiku), and the current frontier models (GPT-5.5, Claude Opus 4.8). EQ-D column is the matched baseline (t1) wave, all-stems; the scoreable subset gives the same ranking (ID .33 vs. $\leq .12$ for every zero-shot model, frontier models ≈ 0). ID grand-average across waves is .36. Enabling reasoning, or few-shot prompting with six anchored exemplars, leaves the two frontier models near zero (Opus 4.8 .06, GPT-5.5 .08 few-shot; see text).

Scorer	MPoDT (all)	MPoDT (scoreable)	EQ-D (t1)
Qwen3.5-27B (zero-shot)	.37	.30	.08
Qwen3.6-27B (zero-shot)	.46	.38	.05
base ensemble (untuned)	.42	.33	.07
GPT-4.1-mini (zero-shot)	.37	.33	.13
Claude-3.5-Haiku (zero-shot)	.41	.38	.14
GPT-5.5 (zero-shot, *frontier*)	.48	.46	.08
Claude Opus 4.8 (zero-shot, *frontier*)	.21	.23	.02
**ID (LoRA-tuned ensemble)**	.41	**.48**	**.31**

Table S18: ID (fixed) vs. length-only and supervised TF-IDF+Ridge (cross-validated) on text criteria.

Corpus / criterion	n	r_{ID}	r_{length}	$r_{\text{TF-IDF (CV)}}$
WASSA: empathic concern	1860	+.35	+.14	+.36
Grossmann: search-for-resolution	424	+.20	+.12	+.01
MPoDT composite	35	+.41	+.45	+.17
